# Fecal microbiota and concentrations of long-chain fatty acids, sterols, and unconjugated bile acids in cats with chronic enteropathy

**DOI:** 10.3389/fvets.2024.1401592

**Published:** 2024-06-12

**Authors:** Maria Veronica Giordano, Paolo Emidio Crisi, Alessandro Gramenzi, Debora Cattaneo, Luca Corna, Chi-Hsuan Sung, Katherine M. Tolbert, Joerg M. Steiner, Jan S. Suchodolski, Andrea Boari

**Affiliations:** ^1^Department of Veterinary Medicine, University of Teramo, Piano D’Accio, Teramo, Italy; ^2^Endovet Professional Association, Rome, Italy; ^3^Gastrointestinal Laboratory, Department of Small Animal Clinical Sciences, Texas A&M University, College Station, TX, United States

**Keywords:** dysbiosis, microbiota, dysbiosis index, fecal metabolome, chronic inflammatory enteropathy, lipid metabolism, inflammatory bowel disease, low-grade intestinal T cell lymphoma

## Abstract

Feline chronic enteropathies (FCE) are common causes of chronic gastrointestinal signs in cats and include different diseases such as food-responsive enteropathy (FRE), inflammatory bowel diseases (IBD), and low-grade intestinal T-cell lymphoma (LGITL). Although changes in intestinal microbiota and fecal metabolites have been reported in dogs and humans with chronic enteropathy, research in cats has been limited. Therefore, this study aimed to evaluate the fecal microbiota and lipid-related fecal metabolites in cats with FCE to a clinically healthy comparison group (CG). A total of 34 cats with FCE (13 FRE, 15 IBD, and 6 LGITL) and 27 cats in the CG were enrolled in this study. The fecal microbiota was evaluated by the qPCR-based feline Dysbiosis Index (DI). The feline DI in cats with CE (median: 1.3, range: −2.4 to 3.8) was significantly higher (*p* < 0.0001) compared to CG (median: - 2.3, Range: −4.3 to 2.3), with no difference found among the FCE subgroups. The fecal abundances of Faecalibacterium (*p* < 0.0001), *Bacteroides* (*p* < 0.0001), *Fusobacterium* (*p* = 0.0398), Bifidobacterium (*p* = 0.0004), and total bacteria (*p* = 0.0337) significantly decreased in cats with FCE. Twenty-seven targeted metabolites were measured by gas chromatography–mass spectrometry, including long-chain fatty acids (LCFAs), sterols, and bile acids (BAs). Fecal concentrations of 5 of 12 LCFAs were significantly increased in cats with FCE compared to CG. Fecal concentrations of zoosterol (*p* = 0.0109), such as cholesterol (*p* < 0.001) were also significantly increased in cats with FCE, but those of phytosterols were significantly decreased in this group. No differences in fecal BAs were found between the groups. Although no differences were found between the four groups, the fecal metabolomic pattern of cats with FRE was more similar to that of the CG than to those with IBD or LGITL. This could be explained by the mild changes associated with FRE compared to IBD and LGITL. The study showed changes in intestinal microbiota and alteration of fecal metabolites in FCE cats compared to the CG. Changes in fecal lipids metabolites suggest a dysmetabolism of lipids, including LCFAs, sterols, and unconjugated BAs in cats with CE.

## 1 Introduction

Chronic enteropathies are gastrointestinal conditions characterized by persistent or recurrent gastrointestinal signs lasting at least 3 weeks, after the exclusion of extra-intestinal, infectious, obstructive, or localized neoplastic causes of gastrointestinal signs (1). Feline chronic enteropathy (FCE) is an umbrella term that includes food-responsive enteropathy (FRE), inflammatory bowel diseases (IBD) and low-grade intestinal T-cell lymphoma (LGITL, previously named alimentary small cell lymphoma, SCL) (2, 3). While diagnosis of FRE is often straightforward due to its clinical resolution with dietary intervention, distinguishing between IBD and LGITL poses challenges due to the overlapping of anamnestic, clinical, and laboratory data (4).

The etiology of FCE remains poorly understood, yet it is acknowledged as a multifactorial condition involving genetic, environmental, and immunological factors, and the intestinal microbiome (5, 6). Changes in intestinal microbiota are observed in cats with chronic diarrhea (7) and with CE (8–6).

The term dysbiosis is continuously evolving, and currently, it refers to changes related to the diversity and/or structure of the intestinal microbiota and its functions (e.g., altered production of metabolites) during pathology (8). This shift in bacterial populations can contribute to the development or exacerbation of gastrointestinal disease. Recently, a dysbiosis index (DI) for cats was developed using qPCR assays and was adopted in cats with IBD and LGITL (6). This index measures both the total bacterial and the abundance of beneficial bacterial taxa, such as *Bacteroides*, *Bifidobacterium*, *Clostridium* (*Peptacetobacter*) *Hiranonis*, *Faecalibacterium*, and *Turicibacter*, as well as taxa that are typically overrepresented in cats with FCE, such as *E. coli* and *Streptococcus*. It is expressed as a single number where a value below zero is indicative of normal microbiota (6). This promising approach may allow for tracking patients’ responses to treatment and facilitates the comparison of results across studies.

The alterations of intestinal microbiota are associated with changes in metabolites derived from microorganisms. Fecal metabolites provide a thorough insight into gastrointestinal health, elucidating the intricate interplay among host metabolism, diet, and gut microbiota activities (9, 10).

Changes in bile acids, amino acids, and short- and long-chain fatty acids (SCFA and LCFA) were observed in gastrointestinal diseases both in humans (11, 12) and dogs (13, 14). In recent years, research has focused on lipid metabolites, that appear to play a crucial role in GI disease. Short-chain fatty acids (SCFAs), long-chain fatty acids (LCFA), bile acids (BA), and sterols are among the lipid metabolites that have been investigated in veterinary gastroenterology up to date (8, 15). However, research on the fecal metabolome in cats with CE remains limited (15, 16). An untargeted metabolomic study in cats with IBD and LGITL revealed increased concentrations of several amino acids, arachidonic acid, simple sphingolipids, and reduced concentrations of indole derivatives found in their fecal samples compared to healthy cats (16). Only more recently, the first targeted metabolomic study has been conducted in cats, revealing significant differences in fecal lipid metabolites between cats with IBD and LGITL compared to healthy cats. However, to date, no data are available for cats with FRE (15). Studies in this area are limited, and further research is needed to elucidate their role more comprehensively, especially in all classes of FCE.

We hypothesize that all classes of FCE, including FRE cats, may exhibit alteration in both the intestinal microbiome and the fecal metabolome compared to clinically healthy cats. Therefore, our study aimed to investigate changes in gut microbiota in cats with CE by assessing the Dysbiosis Index (DI) using a validated qPCR assay (6) and to explore potential alterations in fecal lipids between cats with FCE and a clinically healthy comparison group (CG), using a targeted metabolomics assay.

## 2 Materials and methods

### 2.1 Study population and diagnostic investigations

This study was an observational cohort study. Cats admitted to the Veterinary Teaching Hospital (VTH) of the University of Teramo between January 2020 and November 2022 were prospectively enrolled in the study. All cats were client-owned and were fed various commercial pet foods or home-prepared diets.

Cats with persistent or intermittent clinical signs of chronic enteropathy (e.g., weight loss, hyporexia, vomiting, diarrhea) lasting for at least 3 weeks, were eligible for enrollment in the group of cats with chronic enteropathy (FCE) (17).

Extra-gastrointestinal disease were excluded based on history, physical examination (including a 9-point BCS), a direct fecal smear evaluation and zinc sulfate centrifugal flotation and PCR for *T. fetus* if large intestinal diarrhea was present, complete blood count (CBC), serum biochemistry profile, urinalysis, total T4 (for cats >6 years of age), for all abdominal ultrasound, serum feline pancreatic lipase immunoreactivity (fPLI) and serum feline trypsin-like immunoreactivity (fTLI) if indicated by historical findings.

For cats with CE, exclusion criteria were the presence of systemic, or extra gastrointestinal disease.

These cats may not have received recent (< 6 months) antacids, or antibiotic treatment, nor omega-3 supplementation within the last 4 months, to avoid the effects of omega supplementation. This washout period is recommended based on previous studies evaluating the impact of omega supplementation on fecal metabolites (18). In CE cats, when available, serum cobalamin and folates concentrations, and feline CE activity index (FCEAI) (19) were recorded.

Clinically healthy cats without gastrointestinal signs, systemic diseases, chronic illnesses, or clinically significant laboratory abnormalities were assigned to the CG.

Cats affected by CE were classified as having FRE, IBD, and LGITL (3, 19). In particular, the group of FRE included patients who experienced complete remission of gastrointestinal symptoms within 2 weeks of a dietary trial with a single novel protein source or hydrolyzed protein diet (19, 20). Patients who failed to respond to at least two dietary trials underwent gastro-duodenoscopy and ileo-colonoscopy for diagnostic purposes. When an underlying LGITL was suspected by the pathologist based on histopathology, additional CD3 and CD20 immunohistochemical staining and clonality test (21) were performed to confirm the diagnosis. A final diagnosis of IBD or LGITL was reached by integrating the results from histopathology, immunohistochemistry, and clonality test based on the current guidelines for interpretation and reporting of Ig/TCR clonality testing in suspected lymphoproliferation (3). The same therapeutic diet was continued throughout the trial in all FCE cats.

### 2.2 Sample collection and processing

At the time of the first visit, for each cat included in the study one fecal sample was collected after spontaneous defecation by the owners. Owners were instructed to collect naturally passed feces at home within 24 h of defecation, a period that has been previously shown to have only a minimal effect on microbiome structure in cats at ambient temperatures (22). Collected fecal samples were frozen (−20°C) immediately after collection and shipped to the Gastrointestinal Laboratory at Texas A&M University on dry ice (22, 23). Upon arrival, fecal samples were immediately divided into aliquots and stored at −80°C until analysis. An overnight frozen (−80°C) fecal sample was subsequently transferred into a lyophilizer for freeze-drying for targeted metabolomic analysis. Before DNA extraction for DI, an aliquot of the frozen fecal sample was thawed at room temperature for approximately 2 h. Written informed client consent was obtained before the enrollment of each cat.

### 2.3 Feline dysbiosis index

Fecal samples from client-owned cats in the CG and cats with FCE were collected and quantitative PCR (qPCR) assays were utilized to measure the fecal abundance of total bacteria and seven bacterial taxa: *Bacteroides*, *Bifidobacterium*, *C. hiranonis*, *Escherichia coli*, *Faecalibacterium*, *Streptococcus*, and *Turicibacter*. A dysbiosis index (DI) based on these qPCR abundances as previously described (6) was calculated.

### 2.4 Targeted metabolomic analysis of fecal samples

A targeted validated metabolomic approach in lyophilized fecal samples was utilized to measure the concentrations of unconjugated fecal primary BAs [cholic acid (CA) and chenodeoxycholic acid (CDCA)] and secondary BAs lithocholic acid (LCA), deoxycholic acid (DCA), and ursodeoxycholic acid (UDCA), 12 long-chain fatty acids (LCFAs), and 10 sterols using gas chromatography coupled with a mass spectrometry protocol (GC–MS), as previously described (15). Fecal concentrations of BA were expressed as ng/mg of lyophilized feces, as well as the percentage of total BA. Fecal concentrations of LCFAs and sterols were expressed as μg/mg of lyophilized feces.

### 2.5 Statistical analysis

All datasets were tested for normal distribution using the Shapiro–Wilk test. Comparisons of sex and breed between cats with CE and CG cats were evaluated using Fisher’s exact tests. Comparisons of qPCR bacterial abundance of bacterial taxa and fecal concentrations of each metabolite in cats with FCE and CG cats were compared using the Student’s t-test or Mann–Whitney U-test as appropriate. One-way ANOVA was used to compare the DI and each bacterial taxon between CG cats, cats with FRE, cats with IBD, and cats with LGITL, additionally, it was used to compare the fecal concentrations of each targeted metabolite among these groups. A *Post hoc* comparison with Dunn’s test was used to identify the differences between groups. A power calculation was performed with the software G Power.

The threshold of statistical significance was set at *p* < 0.05. To adjust multiple comparisons, the *p* values were adjusted using a Bonferroni correction. A multiple linear regression analysis was performed to evaluate the associations between the DI, age, FCEAI or serum concentrations of cobalamin, and serum folates. Correlations between the abundance of bacterial groups and fecal concentrations of targeted metabolites were evaluated by Spearman correlation analysis. Statistical analyses were performed using GraphPad Prism 9.0 (GraphPad Software Inc., San Diego, California). Principal coordinate analysis and hierarchical clustering heatmaps were generated using Metaboanalyst 5.0 based on the log-transformed with Pareto scaling data (24). These analyses were performed to compare fecal metabolites between the group and among different age classes in both FCE and CG cats.

## 3 Results

### 3.1 Study population

Sixty-one cats were enrolled in total in the study, with 27 cats included in the CG and 34 in the FCE group. A total of 3 cats were excluded from the FCE group due to concomitant pathologies, specifically 2 with chronic kidney disease and one with hyperthyroidism. Given the numerosity of the groups and assuming probability (1-β error probability) of 0.8 and α of 0.05 the calculated effect size was 0.75.

The characteristics of enrolled cats that underwent fecal microbiota and metabolites analysis are summarized in Tables 1, 2.

**Table 1:** Comparison of demographic data between healthy cats and cats with feline chronic enteropathy CE.

	CG	FCE	*p value*
Number of cats	27	34	
Median age in months (IQ)	45 (5–163)	112.5 (55.5–129)	**0.0001**
Median BW in Kg (IQ)	3.73 (2.2–5.1)	4 (3.2–5.1)	0.2433
Median BCS (IQ)	4 (3–7)	4 (3–5)	0.1340
Sex	3F, 12FS, 5 M, 7MN	1F, 14FS, 4 M, 14MN	0.3872
Breeds	27 DSH	29 DSH, 2 Main coon, 1 Carthusian, 1 British SH, 1 Abyssinian	

BCS body condition score, 1–4 underweight, 5 ideal, 6–9 overweight, BW body weight, DSH domestic shorthair, FS female spayed, MN male neutered.

**Table 2:** Comparison of demographic data between cats in the comparison group (CG) and cats with feline chronic enteropathy CE.

	CG	FRE	IBD	LGITL	*p value*
Number of cats	27	13	15	6	
Median age in months (range)	45 (24–93)	59 (60–128)	120 (60–128)	155 (140.3–162.5)	**0.0001** ^**a**^
Median BW in Kg (IQ)	3.73 (2.2–5.1)	4.3 (3.2–5.3)	4.1 (3.2–5.3)	3.5 (3.2–4.0)	0.2433
Median BCS (IQ)	4 (4–5)	4 (4–5.5)	3 (3–4)	4 (3–5)	**0.0317** ^**b**^
FCEAI	-	2.5 (2–4.7)	10 (8–12)	12 (8.5–13.5)	**0.0004** ^**c**^
Sex	3F, 12FS, 5 M, 7MN	6FS, 1 M, 6MN	1F, 6FS, 2 M, 5MN	2FS, 1 M, 3MN	0.8810
Breeds	27 DSH	12 DSH, 1 Abyssinian	11 DSH, 2 Main coon, 1 Carthusian, 1 British SH	6 DSH	

(FRE: Food-responsive enteropathy; IBD: inflammatory bowel disease; LGITL: Low-grade intestinal T-cell lymphoma), BCS body condition score, 1–4 underweight, 5 ideal, 6–9 overweight, BW body weight, DSH domestic shorthair, FS female spayed, MN male neutered. ^a^ IBD and LGITL cats had a significantly higher age (*p* = 0.0001) compared to CG; ^b^IBD cats had a significantly lower BCS (*p* = 0.0135) compared to CG; ^c^FCEAI was significantly higher in IBD and LGITL compared to FRE.

Among the CG group, 12 were male cats (*n* = 7 neutered) and 15 were female cats (*n* = 12 spayed), while in the FCE group, 19 were male cats (15 neutered) and 15 were female cats (1 intact female). A total of 27 mixed-breed cats were in the CG, and 30 mixed-breed cats and 4 purebred cats (Maine Coon, Abyssinian, Carthusian, and British shorthair) were in the FCE group. The median age was 59 months (range: 5–158 months) in CG cats and 95.2 months in cats with FCE (range: 4–178 months).

Within the FCE group, 13 were classified as FRE, 15 as having IBD, and 6 as having LGITL. The univariate analysis revealed that cats with FCE (*n* = 34) were significantly older (*p* < 0.0001) than CG cats (*n* = 27), particularly for IBD and LGITL cats compared to CG (*p* = 0.0201 and *p* = 0.0002, respectively). However, age was not a significant factor when multiple linear regression was performed (see results below). The median FCEAI score in cats with FCE was 7.45 (range: 1–18) out of a maximum possible score of 18, particularly for IBD and LGITL compared to FRE (for both *p* = 0.0004). The most common clinical signs of cats with CE were weight loss (61%) and vomiting (55%), followed by diarrhea (50%), decreased appetite (41%), and decreased activity/attitude (15%). Eight cats (23.5%) presented with only one of the clinical signs, 14 cats (41.1%) with two signs, and 13 cats (38.2%) with three signs. The clinical findings in cats with CE are summarized in Table 3. At the time of the first visit, all 27 CG cats were fed a commercial maintenance diet and among the cats with CE, six cats were fed a homecooked maintenance diet, while 30 cats were on commercial specialized diets: 10 on a low residual diet (“gastrointestinal diet”), 11 on a hydrolyzed protein diet, and 4 on a hypoallergenic diet. The dietary trial lacked standardization due to the challenges of enforcing a uniform diet for all FCE cats, especially considering their reduced appetite. Among the FRE group, eight responded positively to a commercial hydrolyzed diet and four to a home-cooked diet. Serum folates and serum cobalamin levels were available for 26 out of 34 FCE cats, with median folates levels of 15.2 μg/L (5.9 μg – 58.3 μg/L) (reference range: 9.7–21.0 μg /L for the methods used) and median cobalamin levels of 669.5 ng/L (100 ng/L − 1,648 ng/L) (range 150 ng/L represents the lower detection limit for cobalamin methods used, i.e., chemiluminescence).

**Table 3:** Clinical findings in cats with chronic enteropathy.

Variables	*n* (%)	Total number evaluated
*Gastrointestinal signs*		
Weight loss	21 (61.7%)	34
Vomiting	19 (55.8%)	34
Diarrhea	17 (50%)	34
Decreased appetite	14 (41.1%)	34
Decreased attitude/activity	5 (14.7%)	34
*Clinicopathologic variables*		
Decreased serum cobalamin (< 290 ng/L)	9 (34.6%)	26
Increased serum folate (> 21.6 μg/L)	6 (23.0%)	26
Decreased serum folate (< 9.7 μg/L)	9 (34.6%)	26
Increased dysbiosis index (>0)	27 (77%)	34

### 3.2 qPCR analysis of the fecal microbiota

**Figure 1:**
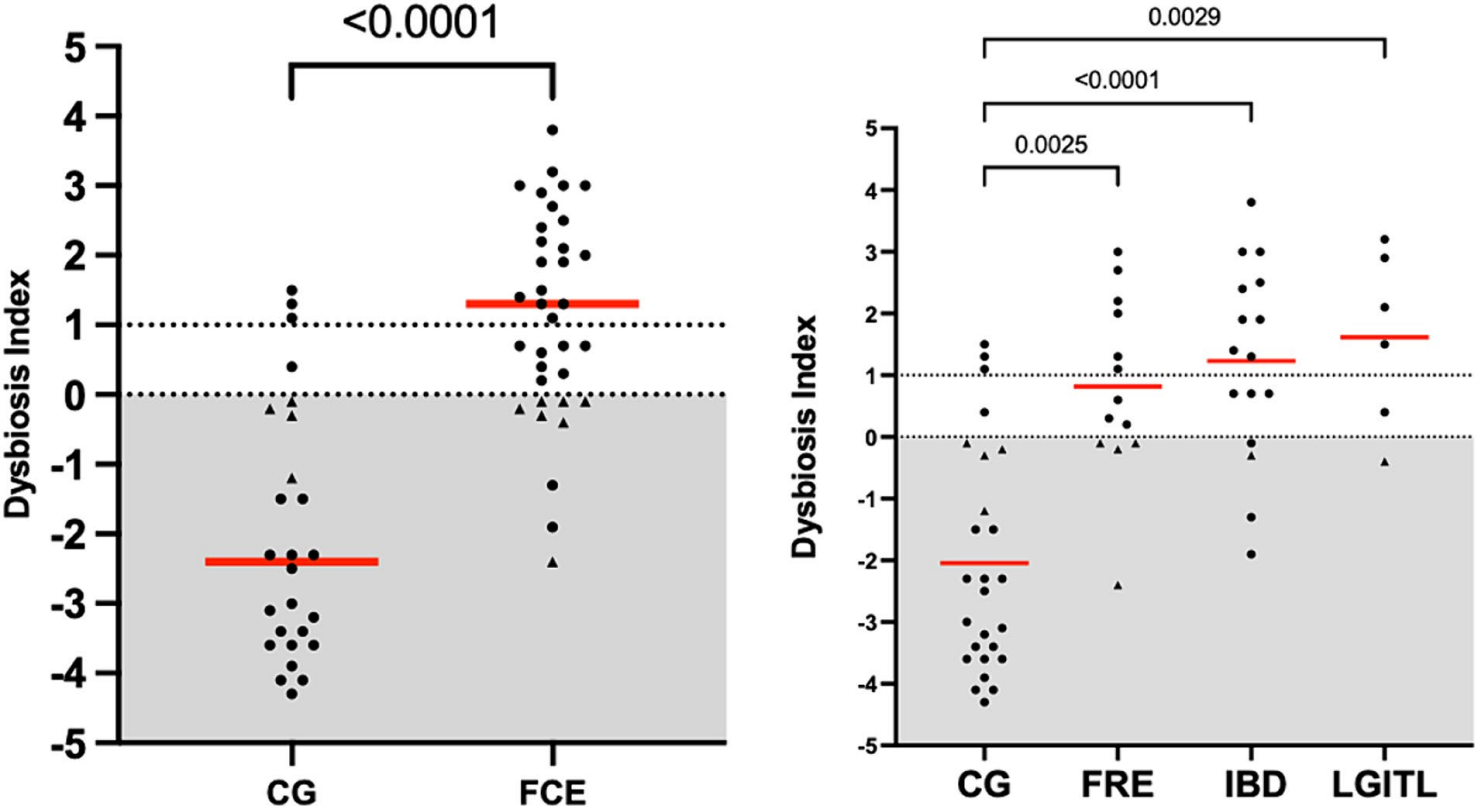
Dysbiosis index in the comparison group (CG) and in cats with FCE; on the right: dysbiosis index in the different groups: cats with food-responsive enteropathy (FRE), inflammatory bowel diseases (IBD), and cats with low-grade intestinal T-cell lymphoma (LGITL). The triangle shapes indicate cats with minor changes (DI < 0, but at least one targeted taxa out of its respective reference interval). Red Horizontal lines represent medians.

**Figure 2:**
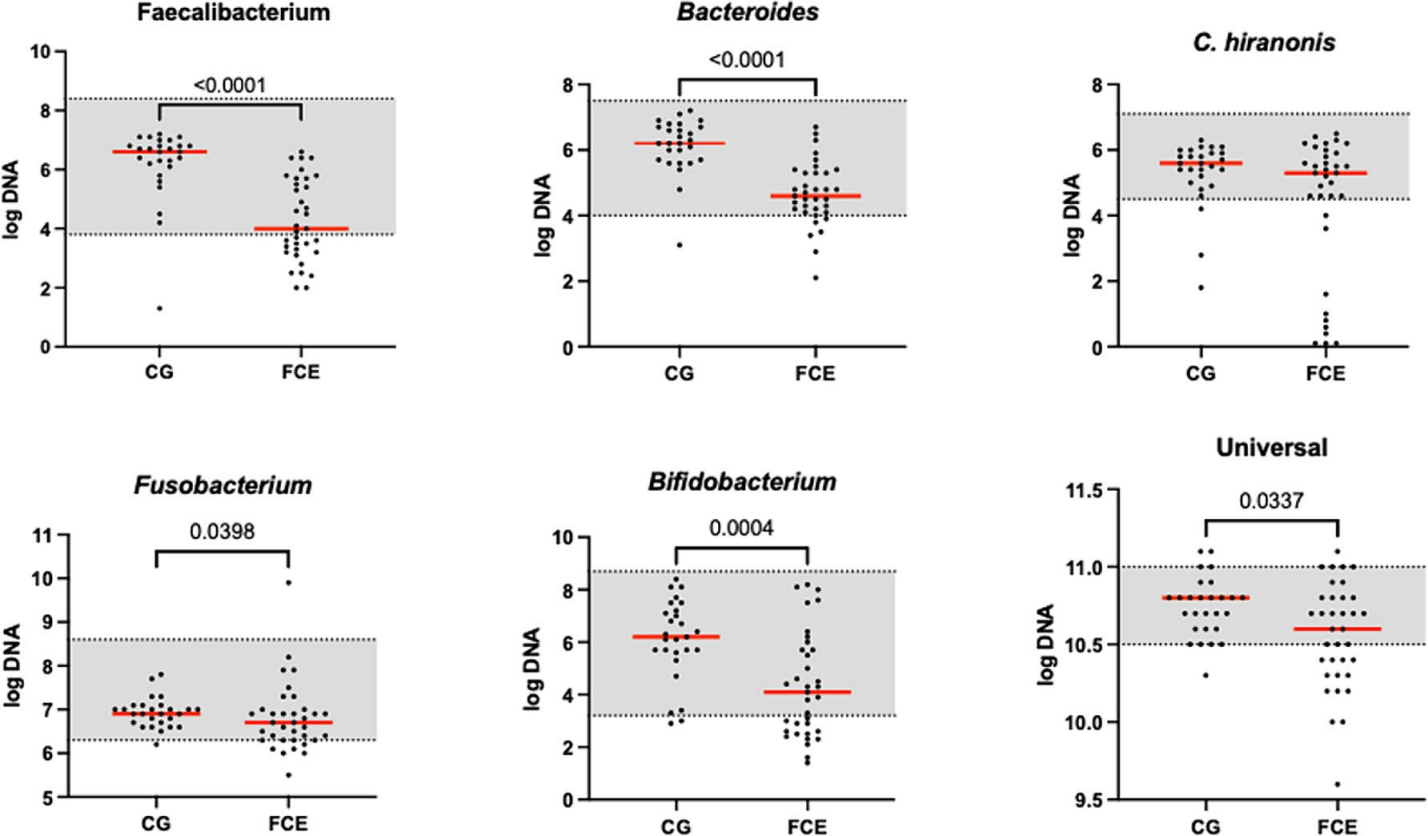
Results of the quantitative PCR panel in the comparison group (CG) and in cats with FCE. The gray area represents the reference interval. Horizontal lines represent medians. Only the significative *p-value* are shown in the graph.

**Figure 3:**
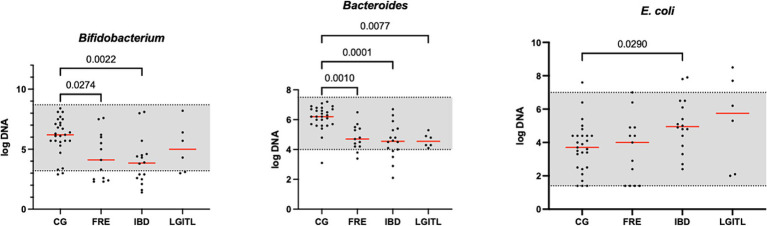
Results of the significative quantitative PCR panel in the comparison group (CG) and cats with food-responsive enteropathy (FRE), inflammatory bowel diseases (IBD), and cats with low-grade intestinal T-cell lymphoma (LGITL). The gray area represents the reference interval. Horizontal lines represent medians. Only the significative *p-value* are shown in the graph.

Figure 1 shows the DI in the CG and in FCE cats as well as the comparison between the DI of the CG with the different FCE groups. Figures 2, 3 show the abundances of each significative bacterial group targeted in the qPCR panel in the CG and FCE and CG compared to the different FCE groups. Twenty-seven out of 34 (77%) FCE cats had DI > 0, while 22 out of 27 (86%) cats in the CG had DI < 0. The feline DI was significantly higher (*p* < 0.0001) in cats with FCE (median: 1.3, range: −2.4 to 3.8) compared to the CG (median: - 2.3, range: −4.3 to 2.3). Although FCE groups differed from the CG, no difference was found among the FCE subgroups. The abundance of *Faecalibacterium* (*p* < 0.0001), *Bacteroides* (*p* < 0.0001), *Fusobacterium* (*p* = 0.0398), *Bifidobacterium* (*p* = 0.0004), and total bacteria (*p* = 0.0337) significantly decreased in cats with FCE, while *E. coli* and *Streptococcus* did not differ significantly between the two groups. The abundance of *C. hiranonis* was below the Reference Intervals (RI) in 10/34 (29.4%) of cats with CE.

**Figure 4:**
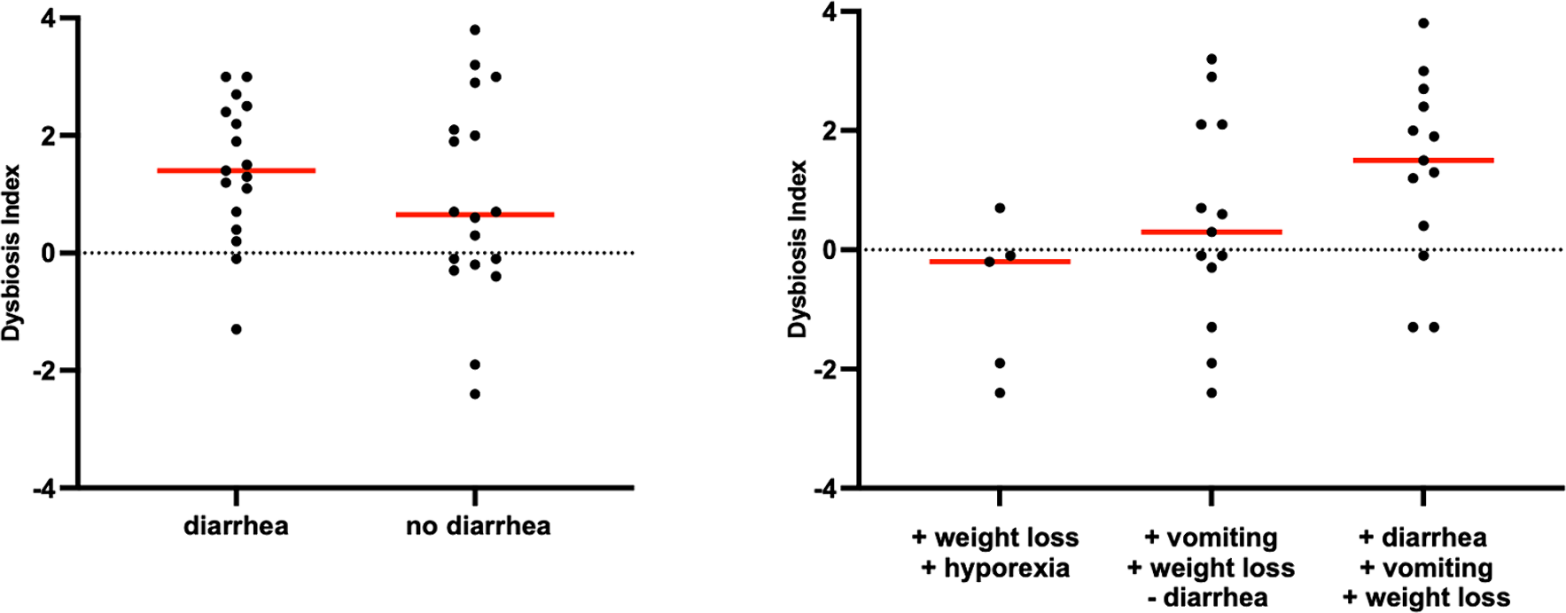
Dysbiosis index in cats with chronic enteropathy separated by main clinical signs. Cats were classified based on the presence or absence of diarrhea.

**Figure 5:**
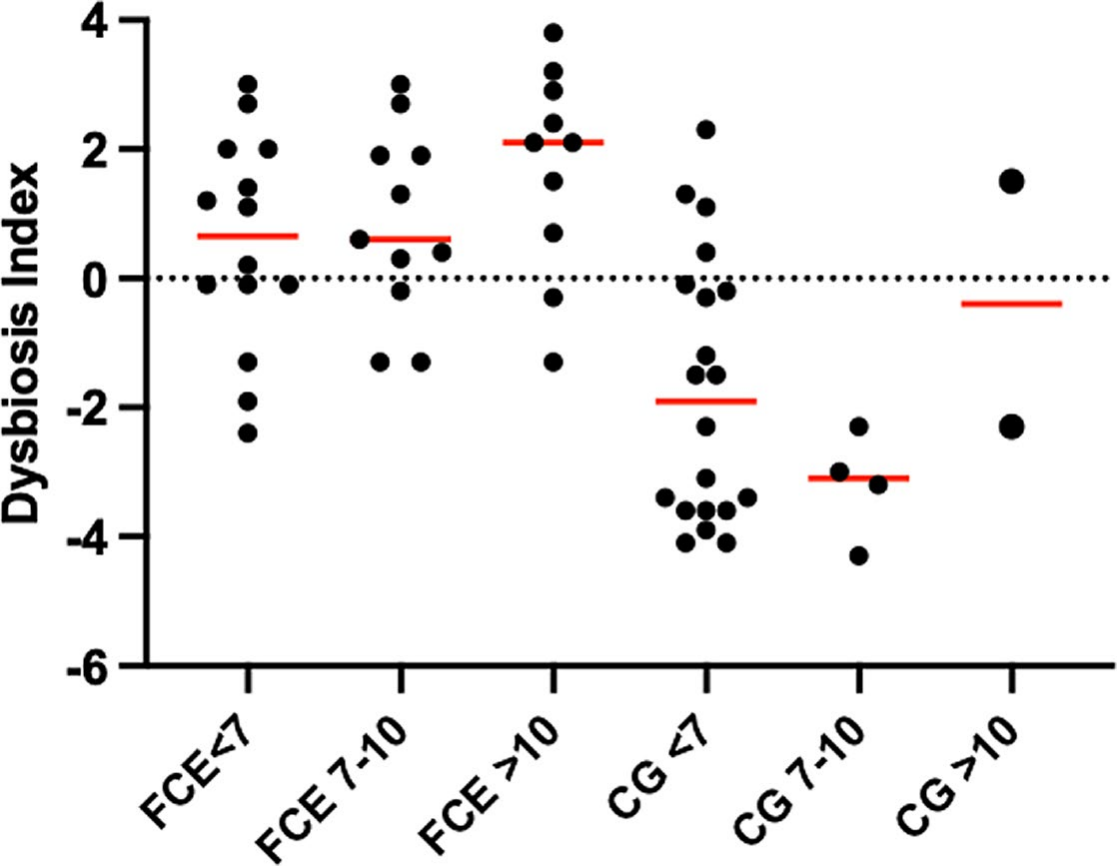
Dysbiosis index (DI) of cats in different age groups. The DI of cats in different age groups was not significantly different within the comparison group (CG; *p* = 2,870 for all) or cats with chronic enteropathy (FCE; *p* = 0.1831 for all).

However, an increase in the abundance of *E. coli* (*p =* 0.0268) was found in cats with IBD compared to the CG (see Figure 3). None of the other targeted bacterial taxa differed between cats with FRE, IBD, and LGITL. No differences were found in DI and clinical signs even if compared with cats with diarrhea and without diarrhea (see Figure 4) or in cats with FCE presenting with different clinical signs. Thirteen cats with FCE exhibited vomiting and weight loss without diarrhea. Among these cats, 7 displayed an increased DI. Thirteen cats displayed vomiting and weight loss associated with diarrhea, of which 9 had an increased DI. Additionally, 3 cats exhibited only diarrhea, all of which had an increased DI. Furthermore, 5 cats showed weight loss and anorexia, of which one had an increased DI. Sixteen cats showed diarrhea among the clinical signs, and among these, 15 had an altered DI. However, of the 18 cats that did not exhibit diarrhea, 11 had an altered DI. No correlation was found between DI and FCEAI. The age of the CG did not match the FCE group. DI did not differ between cats with FCE among the three age groups based on the univariate analysis and multiple regression model (Figure 5).

### 3.3 Fecal concentrations of long-chain fatty acids, sterols, and unconjugated bile acids

**Figure 6:**
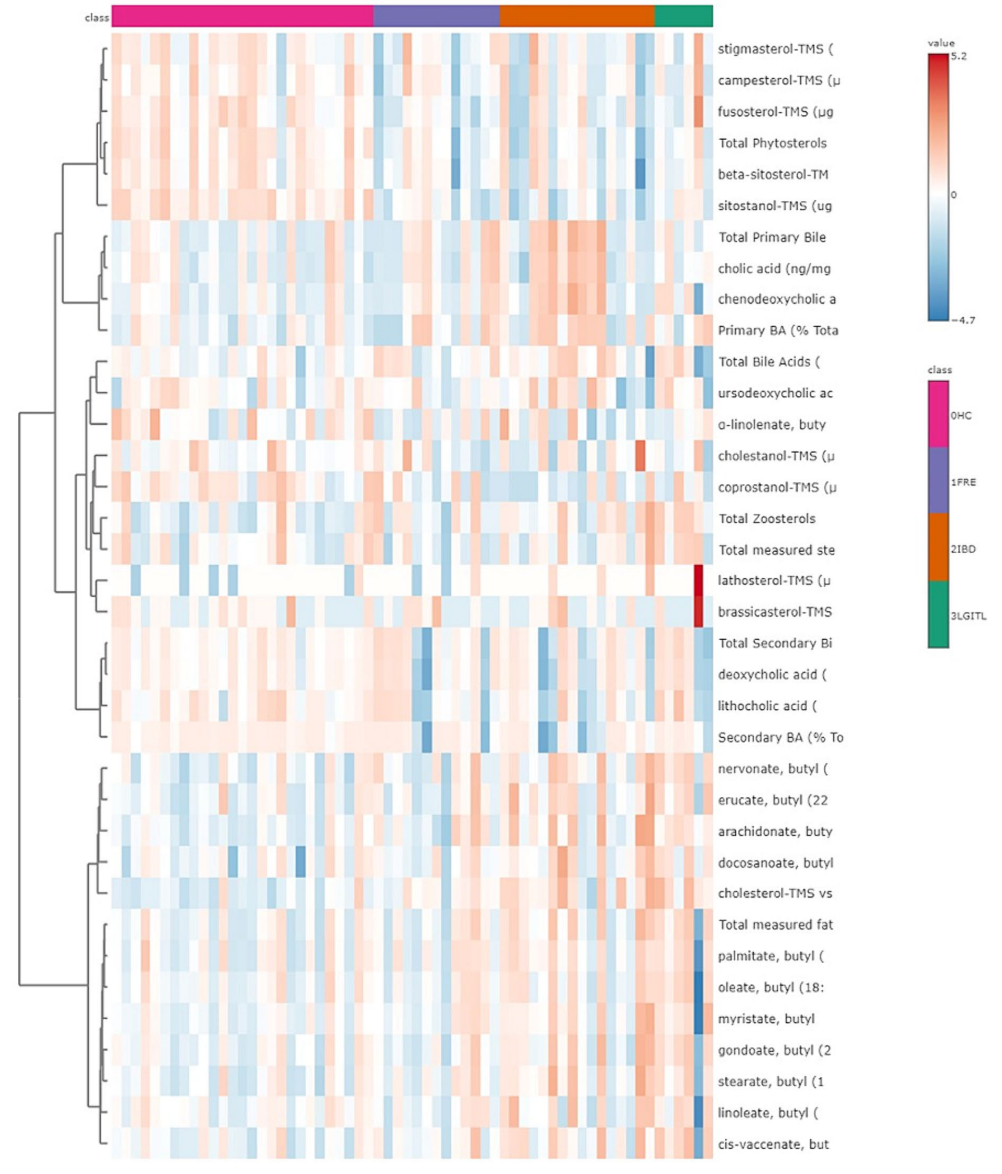
Heatmap of the targeted metabolites in feces. Each row represents the intensity of one metabolite; each column represents one sample. The higher the concentration of a metabolite, the more intensely red shows. The lower the signal intensity of a metabolite, the more intensely blue the metabolite shows in the heat map. Each row corresponds to a single metabolite.

A heatmap, which graphically represents variations in all fecal target metabolites among the four groups of cats, is presented in Figure 6.

**Figure 7:**
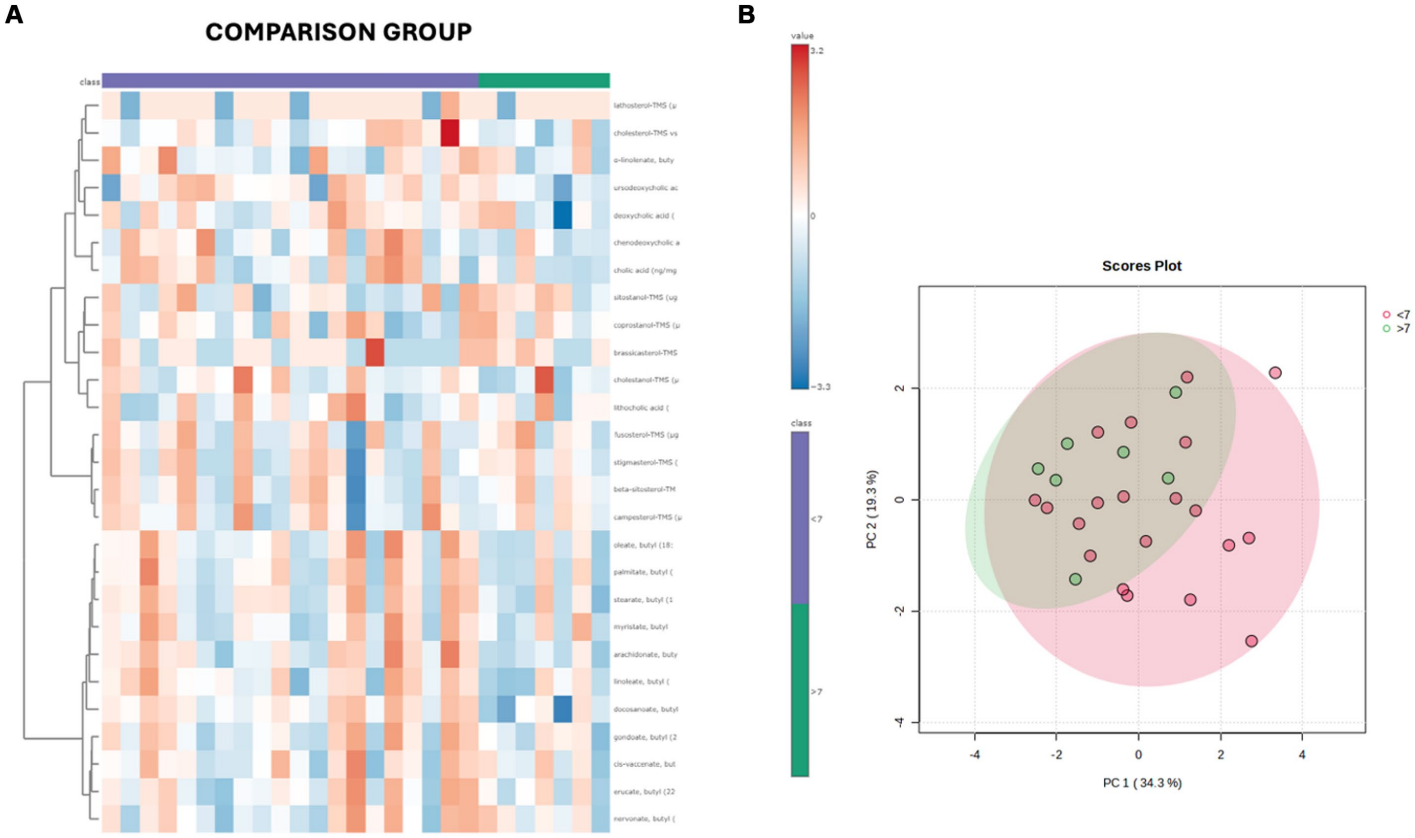
**(A)** Heatmap of the targeted metabolites in feces of cats of different age groups both in CG (*n* = 27). Each row represents the intensity of one metabolite; each column represents one sample. The higher the concentration of a metabolite, the more intensely red shows. The lower the signal intensity of a metabolite, the more intensely blue the metabolite shows in the heat map. Each row corresponds to a single metabolite. Groups of ages are represented by the colored bars at the top of the figure as shown in the legend (violet for <7 years and green >7 years). **(B)** PCA score plots of metabolites in feces from CG. The fecal concentrations of LCFAs, sterols, and BAs did not cluster based on the age group based on a visual examination on the heatmap and PCA plot.

**Figure 8:**
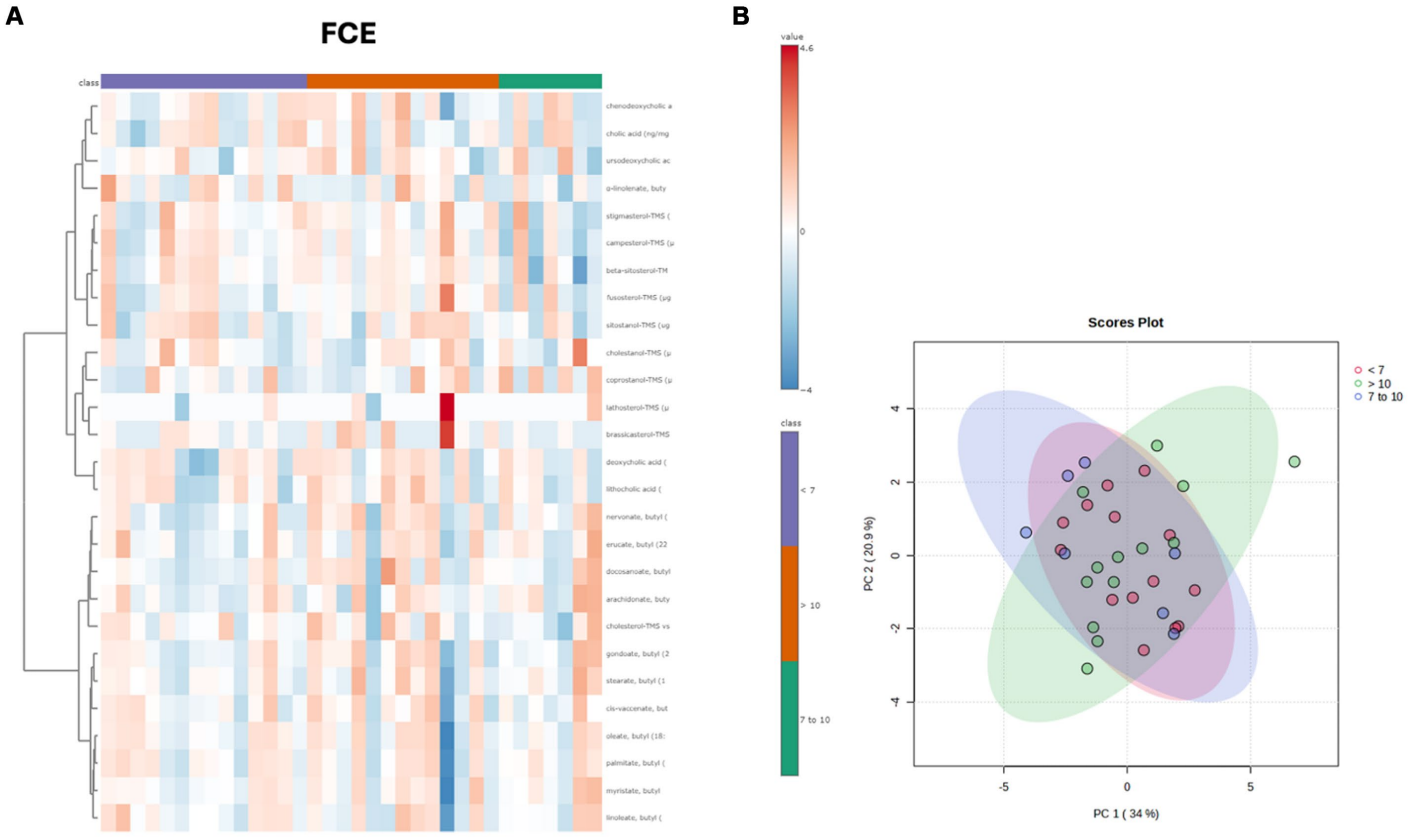
**(A)** Heatmap of the targeted metabolites in feces of cats of different age groups both in FCE cats (*n* = 34). Each row represents the intensity of one metabolite; each column represents one sample. The higher the concentration of a metabolite, the more intensely red shows. The lower the signal intensity of a metabolite, the more intensely blue the metabolite shows in the heat map. Each row corresponds to a single metabolite. Groups of ages are represented by the colored bars at the top of the figure as shown in the legend (violet for <7 years, green from 7 to 10 years and orange >10 years). **(B)** PCA score plots of metabolites in feces from FCE. The fecal concentrations of LCFAs, sterols, and BAs did not cluster based on the age group based on a visual examination on the heatmap and PCA plots.

Both in the CG and FCE, the fecal concentrations of LCFAs, sterols, and BAs did not cluster based on the age group based on a visual examination of the heatmap and PCA (Figures 7A,B, 8A,B).

**Figure 9:**
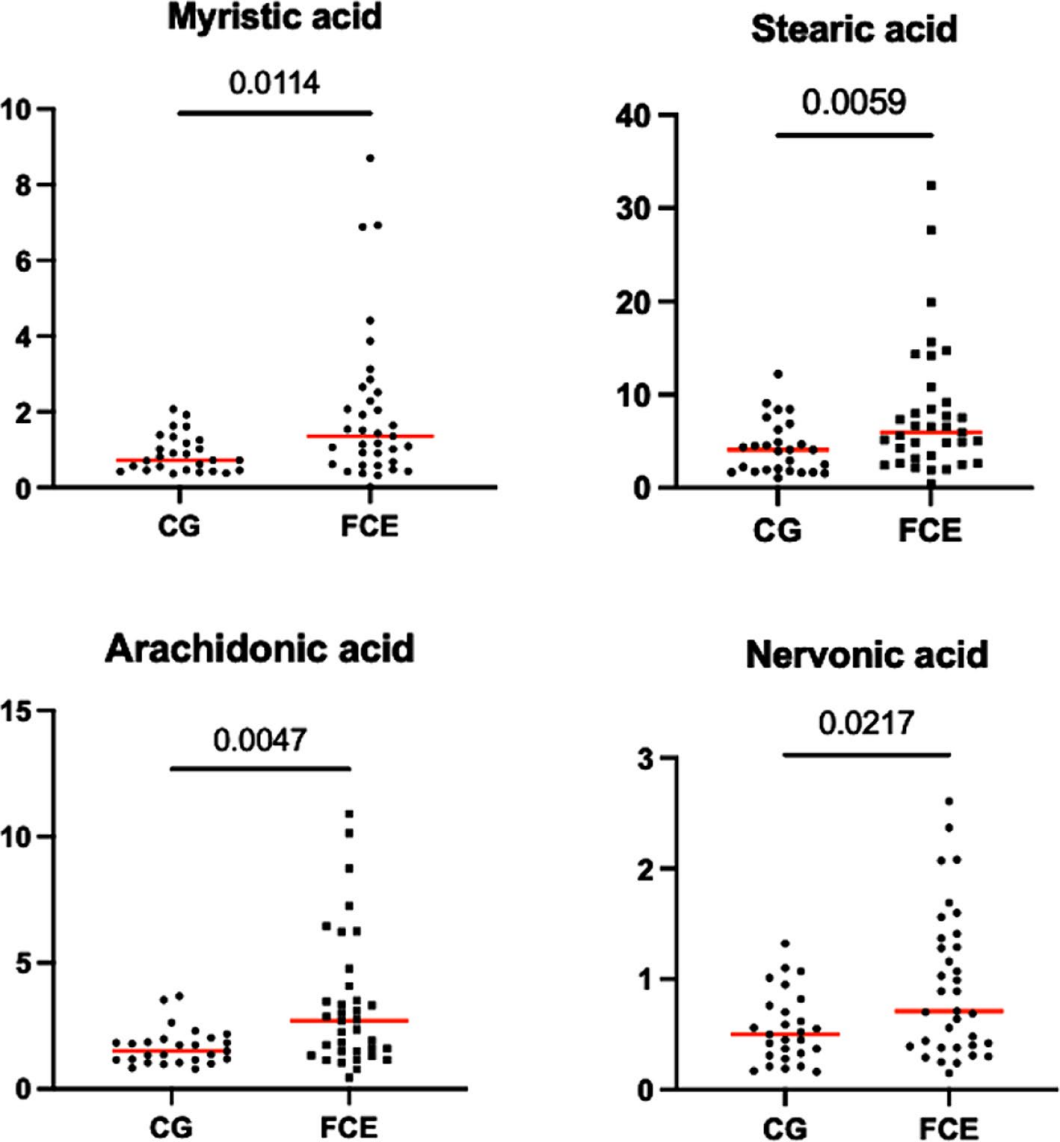
Fecal concentration of the significative fatty acids (μg/mg) in the comparison group (CG) (*n* = 27) and in cats with FCE (*n* = 34). Red lines represent the median. The graphs show the original *p-value*, the corrected one are in the Table 4.

**Figure 10:**
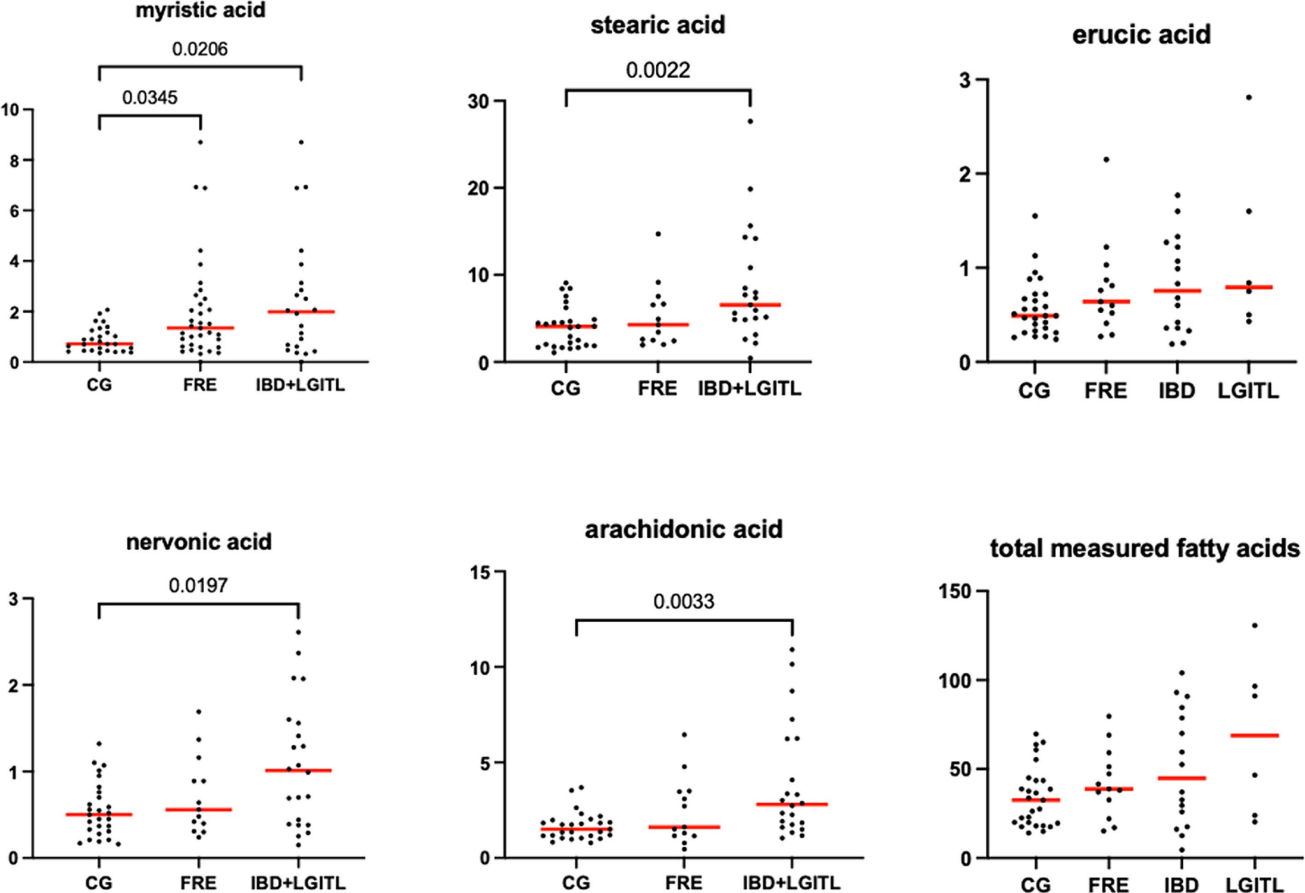
Fecal concentration of fatty acids (μg/mg) in the comparison group (CG) (*n* = 27) and cats with food-responsive enteropathy (FRE, *n* = 13) and cats with IBD and LGITL (*n* = 21) together. Red lines represent the median. The graphs show the original *p-value*, the corrected one are in Table 4.

**Figure 11:**
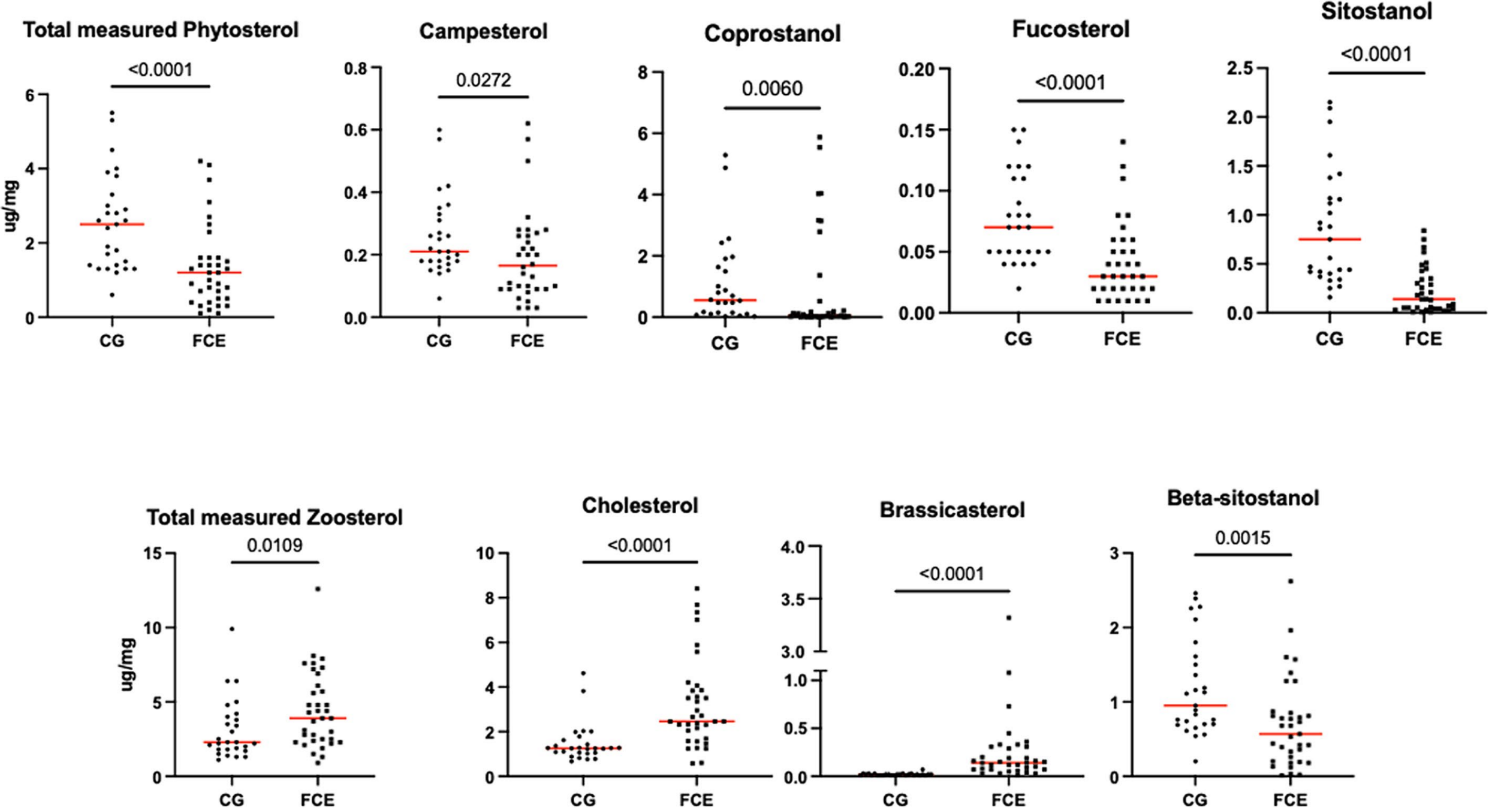
Fecal concentration of sterols (μg/mg) in the comparison group (CG) (*n* = 27) and in cats with FCE (*n* = 34). Red lines represent the median. The concentration of the total measured zoosterol is the sum of coprostanol, cholesterol, cholestanol, and lathosterol. The concentration of the total measured phytosterol is the sum of the rest measured sterols. The *p*-values shown in the figures are unadjusted. For adjusted *p*-values, please refer to Table 4.

**Figure 12:**
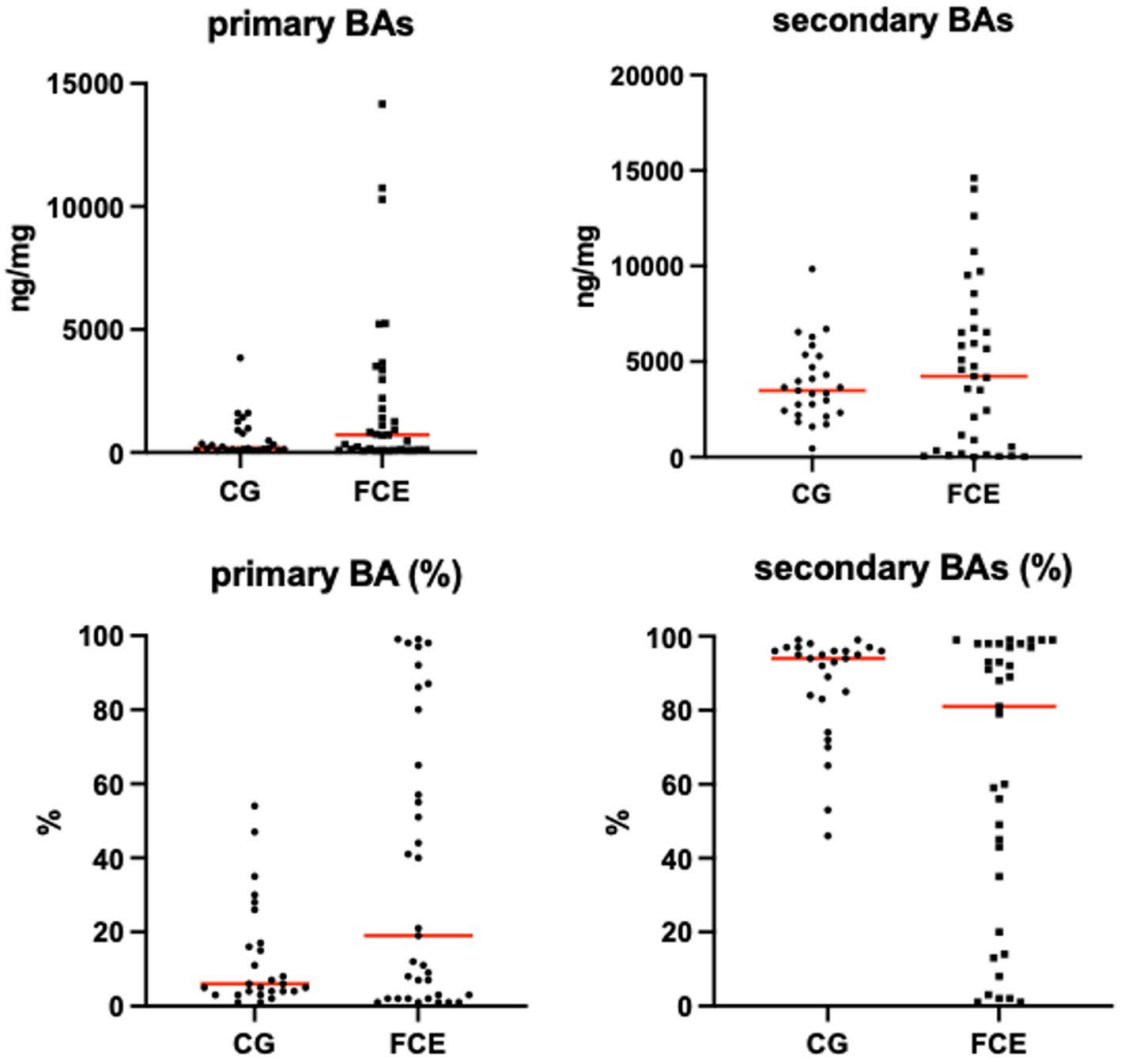
Absolute and relative fecal concentrations of primary and secondary bile acids (BAs) in the comparison group (CG) (*n* = 27) and cats with FCE (*n* = 34). Red lines represent medians. The concentration of primary BAs is the sum of cholic acid and chenodeoxycholic acid. The concentration of secondary BAs is the sum of lithocholic acid, deoxycholic acid, and ursodeoxycholic acid.

Figures 9–12 display the fecal concentrations of the LCFAs, sterols, and BAs in the CG and in cats with CE. The median with minimum and maximum values of each targeted fecal metabolite are reported in Table 4.

**Table 4:** Median values, with minimum and maximum values of fecal metabolites in comparison group and cats with FCE.

Metabolites	Classification	CG (*n* = 27)	FCE (*n* = 34)	*p* value	Adjusted *p* value*
Myristic acid	Fatty Acid	0.72 (0.36–2.07)	1.35 (0.01–8.70)	0.0114^a^	0.1368
Palmitic acid	Fatty Acid	8.21 (3.41–23-4)	11.9(0.60–20.4)	0.0631	0.7572
Stearic acid	Fatty Acid	4.10 (1.09–12-2)	5.95 (0.44–32-4)	0.0059^a^	0.0708
Docosanoic acid	Fatty Acid	0.18 (00.01–0.46)	0.20 (0.04–1.55)	0.1145	1.0
Gondoic acid	Fatty Acid	0.58 (0.18–0.81)	0.65 (0.06–8.74)	0.1663	1.0
Nervonic acid	Fatty Acid	0.50 (0.16–1.32)	0.71 (0.15–2.61)	0.0217^a^	0.2604
Linoleic acid	Fatty Acid	5.76 (2.54–10.5)	5.24 (0.28–20.6)	0.7352	1.0
α-Linoleic acid	Fatty Acid	0.38 (0.12–1.69)	0.20 (0.60–1.73)	0.0661	0.7932
Oleic acid	Fatty Acid	6.58 (2.88–16.8)	7.20 (0.10–20.4)	0.3605	1.0
*Cis*-Vaccenic acid	Fatty Acid	2.66 (0.83–8.69)	3.70 (0.16–17.80)	0.1214	1.0
Arachidonic acid	Fatty Acid	1.50 (0.79–3.68)	2.72 (0.46–10.9)	0.0047^a^	0.0564
Erucic acid	Fatty Acid	0.49 (0.24–1.55)	0.75 (0.19–2.81)	0.0346^a^	0.4152
Coprostanol	Sterol	0.57 (0.02–8.25)	0.05 (0.01–5.88)	0.0033^a^	0.033^a^
Cholesterol	Sterol	1.25 (0.67–4.62)	2.46 (0.58–8.41)	< 0.0001^a^	0.001^a^
Cholestanol	Sterol	0.16 (0.08–0.8)	0.14 (0.03–3.32)	0.1638	1.0
Brassicasterol	Sterol	0.02 (0.01–0.07)	0.14 (0.01–3.32)	< 0.0001^a^	0.001^a^
Lathosterol	Sterol	0.01 (0.00–0.02)	0.01 (0.00–0.61)	0.0372^a^	0.372
Campesterol	Sterol	0.21 (0.06–0.6)	0.17 (0.03–0.87)	0.0498^a^	0.498
Fucosterol	Sterol	0.070 (0.02–0.15)	0.03 (0.01–0.56)	0.0002^a^	0.002^a^
β-Sitostanol	Sterol	0.95 (0.20–2.46)	0.57 (0.01–2.62)	0.0015^a^	0.015^a^
Sitostanol	Sterol	0.75 (0.26–2.15)	0.14 (0.01–0.84)	<0.0001^a^	0.001^a^
Stigmasterol	Sterol	0.17 (0.03–0.31)	0.17 (0.03–0.86)	0.6848	1.0
Cholic acid	pBA	63.0 (5.00–3,125)	302 (1–11,995)	0.2224	1.0
Chenodeoxycholic acid	pBA	152 (62–725)	272 (6–2,895)	0.0544	0.544
Lithocholic acid	sBA	268 (14–1,376)	195 (3–2,708)	0.1114	1.0
Deoxycholic acid	sBA	3,203 (343–8,905)	3,974 (2–12,229)	0.6724	1.0
Ursodeoxycholic acid	sBA	1.00 (0.00–3.00)	0.00 (0.00–22.0)	0.1892	1.0
Primary BA	BA	175 (77–3,850)	721 (78–14,167)	0.1685	1.0
Secondary ΒA	BA	3,481 (444–9,841)	4,241 (12–14,614)	0.7997	1.0
Total BA	BA	4,106 (535–10,743)	5,374 (138–17,747)	0.885	1.0
Primary BA (%)	BA	6.00 (1.00–54.0)	19.0 (1.00–99.00)	0.928	1.0
Secondary BA (%)	BA	95.0 (46.0–99.00)	81.0 (1.00–99.0)	0.928	1.0

^a^Indicate significant differences (^a^*p* < 0.050). Adjusted *p value* ^*^after Bonferroni correction. Primary BA: pBA; Secondary ΒA: sBA.

Fecal concentrations of the significative selected LCFAs of the CG and cats with FCE are shown in Figures 9, 10.

Fecal concentrations of 5 of 12 targeted long-chain FAs were significantly increased in cats with FCE compared to CG cats: myristic acid (*p* = 0.0114; Adj *p* = 0.1368), stearic acid (*p* = 0.0059; Adj *p* = 0.0708), arachidonic acids (*p* = 0.0047; Adj *p* = 0.0564), erucic acid (*p* = 0.0346; Adj *p* = 0.4152), and nervonic acid (*p* = 0.0217; Adj *p* = 0.2604). Fecal concentrations of palmitic acid, oleic acid, *cis*-vaccenic acid, linoleic acid, a-linoleic acid, 11-eicosanoic acids and docosanoic acid did not differ between CG and cats with FCE. Fecal concentrations of selected and significative sterols of CG and cats with FCE are shown in Figure 11.

Fecal concentrations of cholesterol (*p* < 0.001; Adj *p* = 0.001), zoosterol (*p* = 0.0109), brassicasterol (*p* < 0.001; Adj *p* = 0.001), and lathosterol (*p* = 0.0372; Adj *p* = 0.372) were significantly increased in cats with FCE, but those of coprostanol (*p* = 0.0033; Adj *p* = 0.033), campesterol (*p* = 0.0498; Adj *p* = 0.498), fucosterol (*p* = 0.002; Adj *p* = 0.002), beta-sitostanol (*p* = 0.0015; Adj *p* = 0.015), sitostanol (*p* < 0.0001; Adj *p* = 0.001) and phytosterol (*p* < 0.000) were significantly decreased in this group compared to the CG. The ratio of cholesterol/coprostanol was also investigated and resulted significant increase in FCE cats (*p* = 0.0003) compared to the CG.

Fecal concentrations of targeted bile acids in the CG and in cats with FCE are shown in Figure 12. Fecal concentrations of the targeted unconjugated BAs and total measured BAs did not differ between groups. A subgroup of cats with CE (33.3%, n = 12) had increased primary BAs (sum of CA and CDCA divided by total measured BAs) and decreased secondary BAs (sum of LCA, DCA, and UDCA divided by total measured BAs) in feces. No correlation was found between the fecal abundance of *C. hiranonis* and the percentage of primary BA. The percentage of primary BA was calculated as the sum of CA and CDCA divided by the sum of 5 targeted BA.

In a total of 60 cats, the fecal abundance of genus *Bacteroides* and fecal concentrations of cholesterol were negatively correlated [*r* = −0.27, 95% CI (−0.50, −0.02), *p* = 0.0279]; and fecal abundance of *Faecalibacterium* and fecal concentration of sitostanol showed a moderate positive correlation [*r* = 0.60, 95% CI (0.41, 0.74), *p* < 0.0001]. Although the trends were noted, after Bonferroni correction, statistically significant differences were only found in coprostanol (*p* = 0.033), cholesterol (*p* = 0.001), brassicasterol (*p* = 0.001), fucostanol (*p* = 0.002), sitostanol (*p* = 0.001), and beta-sitostanol (*p* = 0.015). No differences were found in the fecal concentration of the target metabolites between cats with FRE, IBD, and LGITL.

## 4 Discussion

The results of this study support the hypothesis that cats with FCE exhibit significant changes in intestinal microbiota and fecal metabolites compared to the CG. Changes in fecal metabolites may reflect functional alterations in the host as well as microbiota and could potentially contribute to a better understanding of gastrointestinal tract functionality.

This study identified a high proportion of dysbiotic cats, with an abnormal DI (DI > 0) observed in 77% of cats with FCE, in contrast to the CG where the DI was within the normal range (DI < 0) in 86% of cases. These results were consistent with those found in previous studies using sequencing methods in dogs and cats with CE (25, 26). Since 14% of clinically healthy cats exhibited an altered DI, we deemed it more appropriate to refer to this group as the comparison group. It is not yet clear whether these clinically healthy cats with subclinical dysbiosis may develop CE in the future. Therefore, further studies in this direction are needed.

In both the CG and FCE cats there were no significant differences observed in the DI (see Figure 5) and fecal metabolites (Figures 7A,B, 8A,B) among various age groups. On univariate analysis, age did not show a correlation with the DI when considering the entire cohort of all CG cats and cats with FCE. This lack of correlation was likely due to the significantly older age of cats with CE compared to those in the CG. However, the DI did not differ between cats with FCE among the three age groups based on the univariate analysis and multiple regression model, as well as the fecal concentrations of LCFAs, sterols, and BAs did not cluster based on the age group based on a visual examination on the heatmap and PCA.

The study’s small sample size, particularly in the LGITL group, posed a limitation as it significantly reduced the power of our analysis. Additionally, the age of the CG did not match the FCE group and the CG comprised younger cats compared to those in the FCE group.

This age difference might have impacted the DI results, given that the fecal microbiome and metabolome can undergo age-related changes age (27, 28), but this study did not investigate the impact of this factor, which represents another limitation of this study. However, when comparing the extent of shifts from biological (age) to disease status, it’s evident that disease should drive more significant shifts and changes (29, 30).

Additionally, there was no observed correlation in DI with sex, FCEAI, body weight, BCS, cobalamin, or folates.

In line with previous studies in cats (5, 6, 23), weight loss was the primary clinical sign observed in our cats with FCE. This observation differs from dogs, where diarrhea is the predominant presenting clinical sign. This finding is not unexpected given the common involvement of the small intestine and consequential malabsorption in FCE compared to the CG, as previously hypothesized in other studies (6). The absence of significant variations in DI between cats exhibiting diarrhea and those that did not suggest that dysbiosis may be present in feline patients with FCE in the absence of specific clinical manifestations. This observation highlights the diagnostic potential of DI in identifying dysbiosis in patients with no evident diarrhea or other gastrointestinal clinical manifestations.

In this study, DI was assessed for the first time in cats with FRE, revealing a higher DI compared to the CG. Previous investigations utilizing 16S rRNA gene sequencing on fecal samples from dogs and cats with FRE indicated baseline microbiota alterations in these animals (31, 32). In the previous sequencing study dietary intervention in cats resulted in an improved microbiota structure, especially in the responder group, after hydrolyzed dietary trials (31). An increase in the abundance of the genus *Bifidobacterium* was associated with improvement, whereas *Oscillibacter* and *Desulfovibrionaceae*_unclassified bacteria were more abundant in non-responders at diagnosis. The authors suggest that these microbial indicators may serve as potential markers for non-response to the diet at diagnosis. A future 16S rRNA gene sequencing study on our samples could be valuable for identifying prevalent bacteria at the time of diagnosis in non-responders cats to diet, thereby confirming the findings of this previous study. However, it has been suggested that the diet might have induced clinical remission through mechanisms not directly related to the microbiome, implying that observed changes in the microbiota could be a consequence rather than the primary cause of remission (31). In pediatric patients with Crohn’s disease, variations in the composition of the fecal microbiota were observed between individuals responding and those not responding to dietary therapy at baseline (33). This highlights the potential of the microbiota as early predictors of response to dietary therapy.

However, unlike the aforementioned studies, the DI did not exhibit differences between our responder (FRE) and non-responder cats (IBD-LGITL) to the diet. The relatively smaller number of cats in our IBD and LGITL group might have diminished the statistical power to detect a significant difference.

In cats that do not respond to dietary trials, other factors are involved in the development of more severe diseases such as IBD and LGITL, for which diet alone is not sufficient to manage the condition (31).

In this study, dysbiosis was observed in cats with IBD and LGITL, but there were no significant differences between both conditions. This finding aligns with the results of a previous study using 16S rRNA gene sequencing and qPCR on feline fecal samples (6, 23). Distinguishing between IBD and LGITL in cats poses challenges due to overlapping clinical signs and intestinal mucosal changes. This difficulty may stem from the considerable similarity between the two conditions, their marked inflammatory nature, and the potential for co-occurrence or progression. As a result, the observed dysbiosis in all classes of FCE could potentially reflect a microbiota that reacts to chronic inflammation regardless of the specific nature of the disease. Another consideration is that the fecal microbiome may not precisely represent bacterial communities in the small intestine, typically implicated in feline cases. Investigating the luminal content or the mucosal microbiome might reveal more distinct differences in microbial communities. However, a recent human study has shown substantial overlaps between fecal and mucosal microbiomes in patients with and without IBD, even though human IBD primarily affects the large bowel (34). Nonetheless, investigations into mucosa-adherent bacteria in cats have revealed increased numbers of *Enterobacteriaceae* adherent to the duodenal mucosa in cats with IBD (35), along with a higher quantity of *Fusobacterium* spp. adherent to ileum and colon biopsies in cats with LGITL compared to IBD (36). These findings suggest a potential role for these bacteria in the pathogenesis of these diseases.

The bacterial groups targeted in the DI belong to the primary bacterial taxa that inhabit the healthy feline gastrointestinal tract (13, 23, 37). Similarly, to previous microbiota studies in cats, in this study cats with FCE exhibited a lower abundance of *Faecalibacterium, Bacteroides, Fusobacterium,* and *Bifidobacterium,* as well as total bacteria, compared to the CG. Although differences were observed in the abundance of various bacterial groups between CG and FCE cats, no statistically significant differences were found between the subgroups of FCE.

This study also revealed the alterations in concentrations of fecal FAs, and sterols, in cats with FCE compared with CG. These findings are consistent with previous metabolomic studies in cats (15, 16), dogs (14) and humans (9, 38, 39) suggesting an altered lipid metabolism in cats with CE.

In general, the concentration of a fecal metabolite can increase as a result of increased synthesis or excretion, decreased degradation, or loss in the intestinal lumen, conversely, it can decrease due to reduced synthesis or increased destruction (32).

Fecal concentrations of 5 of 12 targeted LCFA were significantly increased in cats with FCE compared to the CG: myristic acid, stearic acid, arachidonic acid, erucic acid, and nervonic acid. Similar increases in fecal LCFA were observed in dogs with chronic enteropathy, and human IBD patients (10, 40–43). Consistently, untargeted and targeted metabolomic studies in cats also reported elevated levels of fecal LCFAs (6, 16), supporting the hypothesis of lipid imbalance in this species.

A recent lipidomic study revealed differences in FA-erythrocyte membranes in dogs affected by CE compared to healthy ones (44) supporting the hypothesis of lipid imbalance in this condition.

Studies in human patients with IBD revealed a low concentration of monounsaturated (MUFAs) and polyunsaturated (PUFAs) in serum samples of CD patients and pediatric patients with IBD compared to healthy, contrasting with higher levels found in fecal samples (10, 42, 43, 45), similar results have been observed in dogs with FRE (46). This discrepancy suggests malabsorption in these diseases, as the increased loss of FAs in feces may contribute to the reduced serum concentration of FAs. Damage to the intestinal barrier may result in the loss of receptors responsible for the intestinal absorption of fatty acids (FAs), leading to their increased excretion in feces.

Among the MUFAs, nervonic acid (NA) is particularly noteworthy. Derived from oleic acid, NA is a crucial component of the myelin sheath in nerve tissue, since the intestinal gangliosides are located between the submucosal and muscle layers of the intestine, its increased fecal concentration in cats with CE may suggest more extensive or profound damage to the intestinal barrier. Notably, NA levels were significantly higher in the feces of cats with IBD and LGITL compared to the FRE group, which displayed a profile closer to that of the CG. The observed reduction in serum NA, along with ARA and palmitic acid, in patients with IBD (47), supports the hypothesis of increased intestinal loss in the course of CE. A recent study conducted on mice with DSS-induced colitis has revealed the potential role of NA in binding the PPAR-γ, inhibiting the NF-kb reducing the release of pro-inflammatory cytokines, and in promoting the restoration of the intestinal barrier. Therefore, the loss of anti-inflammatory and repair activities mediated by NA may contribute to the perpetuation of the inflammatory process (48).

The role of FA has been investigated in chronic enteropathy. Particularly, arachidonic acid (ARA) is the precursor of pro-inflammatory mediators, such as Prostaglandins E (PGE), that were found higher in IBD human patients with active disease (40). Reduced levels of ARA have been found in the serum of adult CD and pediatric UC patients, respectively (10, 43) together with an increase of ARA in fecal samples of CD patients (40). On the other hand, the key role of ARA in inflammation has also been demonstrated, as it increases the expression of the intracellular adhesion molecule ICAM-1 is increased by ARA, favoring the inflammatory response by recruiting leukocytes (49, 50).

A recent study has provided evidence of an elevated expression of COX-2 in the epithelial cells of feline biopsies with IBD or LGITL, supporting a major activity of this enzyme in this condition, both due to its involvement in prostaglandin production and the processes of intestinal barrier repair (51). Our results showing the increase in ARA, support the presence of an active inflammatory response occurring in the gastrointestinal tract.

In this study, fecal concentrations of 6 out of 12 sterols were found to be decreased in FCE compared to the CG, mostly belonging to the phytosterols group (plant-derived sterols), such as campesterol, fucosterol, β-sitosterol, and sitosterol, except for coprostanol, which was the only zoosterol (animal-derived sterols) found to be decreased in the FCE. On the other hand, most of the sterols that were found to be increased in FCE cats belonged to the zoosterols group, including cholesterol, and lactosterol, except for the brassicasterols, which belong to the phytosterols (4/12 sterols).

These results are only partially consistent with recent studies in dogs that have reported a decreased concentration of β-sitosterol and sitostanol in dogs with CE dogs (26, 52), and are consistent with the results of the previous untargeted study in cats (15, 23).

Sterols play a critical role in maintaining cell structure by forming a significant portion of the cell membrane and regulating its fluidity, integrity, and permeability (53). Phytosterols, which are structurally similar to cholesterol, have been recognized for their potential to reduce cholesterol absorption (54, 55). Since phytosterols cannot be synthesized *de novo* by mammalian hosts, fecal concentrations reflect dietary ingestion and intestinal absorption. The decrease in phytosterols observed in cats with FCE could be attributed to their low dietary intake of plant sterols. This hypothesis found support in a recent study in dogs, which showed that a diet rich in animal proteins led to a reduction in phytosterols and the phytosterol-to-zoosterol ratio in dogs’ fecal samples (26). Interestingly, our cohort of cats with IBD and LGITL displayed a significantly lower phytosterol-to-zoosterol ratio. Given that most cats in CG were on a maintenance diet and diets were similar in FCE groups, it is improbable that dietary factors alone caused the decline in fecal phytosterols and the rise in zoosterols. Other factors, such as anorexia, malabsorption, and severe damage to the intestinal barrier in FCE cats, may contribute to these results. The presence of changes in appetite and anorexia made it challenging to implement a standardized diet in our FCE cat cohort.

The lack of a standardized diet for both groups before enrollment was one limitation of this study. Implementing a standardized, high-quality diet for the comparison group could have enhanced the interpretation of diet-influenced metabolites, but this protocol was not adopted. However, it’s noteworthy that in this study, most cats of the CG were consuming commercial feline maintenance diets. Additionally, a similar pattern of changes in fecal metabolites were observed in the previous metabolomics studies in dogs (52) and cats with CE (15, 16). where subjects also had varied diets and this condition is representative of the common clinical situation.

Nonetheless, further studies in this direction are essential to better comprehend the role of these fecal metabolites. Unabsorbed sterols are metabolized by intestinal bacteria in the colon (54, 55). Cholesterol can be transformed by bacteria in the large intestine, leading to the production of coprostanol, which has poor absorption rates (56). Human patients with Crohn’s disease, ulcerative colitis, or *Clostridium difficile* infection exhibit lower levels of cholesterol-to-coprostanol conversion (38), however elevated fecal excretion of cholesterol and coprostanol has been demonstrated in patients with colon cancer and adenomatous polyps in comparison to normal individuals from both American and Japanese populations, as well as in patients with other gastrointestinal disorders (57). In our study, CE cats exhibited decreased fecal coprostanol levels and increased fecal cholesterol levels, resulting in a significantly higher cholesterol-to-coprostanol ratio in cats with FCE compared to CG, aligned with previous study in cats (15). The increased ratio reflects a lower conversion of cholesterol to coprostanol and indirectly suggests a reduction in the abundance of bacterial species involved in this conversion, such as *Bacteroides*, which seems to be implicated in humans (58).

This study did not reveal any significant differences in the target bile acids between the CG and FCE cats. This finding is in contrast with the observations made in humans and dogs (6, 14, 59, 60), where dysbiosis is often associated with a reduction in secondary bile acids and an increase in primary bile acids due to a decrease in the abundance of bacteria responsible for their conversion, such as *C. hiranonis*. While there was no significant difference in *C. hiranonis* between the CG and FCE cats, it is noteworthy that a reduction of *C. hiranonis* was observed in 29.4% of our FCE cats (*n* = 10/34). These findings are consistent with a previous targeted metabolomic study in cats, where increased total fecal BAs, elevated fecal primary BAs and reduced secondary BAs were observed in 25% of cats with CE (15). Besides dysbiosis, malabsorption may also contribute to this outcome, along with chronic inflammation and shortened villi (61). The majority of BA reabsorption is the result of active transport mediated by an apical sodium-dependent bile acid transporter (ASBT), that are highly expressed in the distal ileum (61) well described in humans (62). In humans with Crohn’s disease and dogs with CE, a decrease in ileal protein expression of apical sodium-dependent bile ASBT is documented (63, 64), and in dogs, ileal ASBT expression is negatively correlated with the increasing severity of inflammatory histopathologic scores (63). Although studies on ASBT expression and distribution in the feline gastrointestinal tract and in cats with CE are lacking, malabsorption is commonly reported in this condition. Moreover, the excess of primary BAs in the colon may increase motility and shorten transit time, reducing the time required for bacterial conversion of primary BAs into secondary BAs (15, 65). Upon examination of the heatmaps depicting fecal metabolites, particularly fatty acids, and sterols, it was evident that the FRE subgroup more resembled the CG than the two other groups. This observation suggests that in the FRE subgroup the severity of the FCE and the damage to the intestinal epithelium are possibly lower compared to IBD and LGITL. Despite these, no significant correlations were found between any targeted compounds and the FCEAI. Additionally, concentrations of targeted metabolites did not show significant differences between cats with IBD and cats with LGITL, aligning with the results of the untargeted and targeted metabolomic studies in cats (15, 16).

The large effect size represents a potential limitation and imposes a careful interpretation. Validation in larger cohorts, and consideration of the practical significance are necessary to ensure that the findings are both reliable and meaningful in real-world settings. Addressing these limitations through future research will help to establish the robustness and applicability of the observed effects.

Another limitation of this study was that different sampling regions might impact the microbiome and metabolomic profiles. Yet, a study in humans found that the impact of sampling regions is minimal (66). In this study, a fecal sample was manually mixed before aliquoting to minimize this impact of biological variation. Finally, we did not conduct follow-up sampling, which could have provided valuable information on the treatment response. Prospective experiments conducted under more controlled conditions, such as a standardized diet, would likely be necessary to address this issue.

## 5 Conclusion

In conclusion, our study confirmed an alteration in fecal microbiota and metabolites in cats with CE compared to cats in the CG. The DI was found to be altered even in cats with FRE and cats with severe forms of FCE. Finally, the observed trend in cats with FCE suggests that alterations in lipid metabolism are similar to those documented in humans, dogs, and cats affected by this condition. However, further studies are necessary to determine the precise function and importance of these metabolites, particularly in the context of dysbiosis-induced changes in the microbiota. Additionally, longitudinal studies that track these changes over time would be valuable for future research.

## Data availability statement

The raw data supporting the conclusions of this article will be made available by the authors, without undue reservation.

## Ethics statement

Ethical approval was not required for the studies involving animals in accordance with the local legislation and institutional requirements because this study did only involve non-invasive procedures on fecal samples. Written informed consent was obtained from the owners for the participation of their animals in this study.

## Author contributions

MG: Writing – review & editing, Writing – original draft, Resources, Project administration, Investigation, Formal analysis, Data curation, Conceptualization. PEC: Writing – review & editing, Resources, Investigation, Data curation. AG: Investigation, Resources, Writing – review & editing. DC: Data curation, Resources, Writing – review & editing. LC: Data curation, Resources, Writing – review & editing. C-HS: Conceptualization, Formal analysis, Investigation, Methodology, Writing – review & editing. KT: Formal analysis, Resources, Writing – review & editing. JMS: Investigation, Resources, Writing – review & editing. JSS: Conceptualization, Formal analysis, Investigation, Methodology, Project administration, Resources, Supervision, Writing – review & editing. AB: Conceptualization, Investigation, Supervision, Writing – review & editing.

## References

[1] Jergens AE. Feline idiopathic inflammatory bowel disease: what we know and what remains to be unraveled. J Feline Med Surg. (2012) 14:445–58. doi: 10.1177/1098612X12451548, PMID: 22736679 PMC10822384

[2] Barrs VR Beatty JA. Feline alimentary lymphoma: 1. Classification, risk factors, clinical signs and non-invasive diagnostics. J Feline Med Surg. (2012) 14:182–90. doi: 10.1177/1098612X12439265 22370860 PMC10822432

[3] Marsilio S Freiche V Johnson E Leo C Langerak AW Peters I. ACVIM consensus statement guidelines on diagnosing and distinguishing low-grade neoplastic from inflammatory lymphocytic chronic enteropathies in cats. J Vet Intern Med. (2023) 37:794–816. doi: 10.1111/jvim.16690, PMID: 37130034 PMC10229359

[4] Marsilio S. Feline chronic enteropathy. J Small Anim Pract. (2021) 62:409–19. doi: 10.1111/jsap.13332 33821508

[5] Inness VL McCartney AL Khoo C Gross KL Gibson GR. Molecular characterisation of the gut microflora of healthy and inflammatory bowel disease cats using fluorescence in situ hybridisation with special reference to Desulfovibrio spp. J Anim Physiol Anim Nutr. (2007) 91:48–53. doi: 10.1111/j.1439-0396.2006.00640.x 17217390

[6] Sung CH Marsilio S Chow B Zornow KA Slovak JE Pilla R. Dysbiosis index to evaluate the fecal microbiota in healthy cats and cats with chronic enteropathies. J Feline Med Surg. (2022) 24:e1–e12. doi: 10.1177/1098612X221077876, PMID: 35266809 PMC9160961

[7] Suchodolski JS Foster ML Sohail MU Leutenegger C Queen EV Steiner JM. The fecal microbiome in cats with diarrhea. PLoS One. (2015) 10:e0127378. doi: 10.1371/journal.pone.0127378, PMID: 25992741 PMC4437779

[8] Suchodolski JS. Analysis of the gut microbiome in dogs and cats. Vet Clin Pathol. (2022) 50:6–17. doi: 10.1111/vcp.13031 34514619 PMC9292158

[9] Sinha R Ahn J Sampson JN Shi J Yu G Xiong X. Fecal microbiota, fecal metabolome, and colorectal Cancer interrelations. PLoS One. (2016) 11:e0152126. doi: 10.1371/journal.pone.0152126, PMID: 27015276 PMC4807824

[10] Daniluk U Daniluk J Kucharski R Kowalczyk T Pietrowska K Samczuk P. Untargeted metabolomics and inflammatory markers profiling in children with Crohn’s disease and ulcerative colitis-a preliminary study. Inflamm Bowel Dis. (2019) 25:1120–8. doi: 10.1093/ibd/izy402, PMID: 30772902

[11] Marchesi JR Holmes E Khan F Kochhar S Scanlan P Shanahan F. Rapid and noninvasive metabonomic characterization of inflammatory bowel disease. J Proteome Res. (2007) 6:546–51. doi: 10.1021/pr060470d, PMID: 17269711

[12] Ma C Vasu R Zhang H. The role of long-chain fatty acids in inflammatory bowel disease. Mediat Inflamm. (2019) 2019:1–10. doi: 10.1155/2019/8495913 PMC6874876 31780872

[13] Minamoto Y Otoni CC Steelman SM Büyükleblebici O Steiner JM Jergens AE. Alteration of the fecal microbiota and serum metabolite profiles in dogs with idiopathic inflammatory bowel disease. Gut Microbes. (2015) 6:33–47. doi: 10.1080/19490976.2014.997612 25531678 PMC4615558

[14] Blake AB Guard BC Honneffer JB Lidbury JA Steiner JM Suchodolski JS. Altered microbiota, fecal lactate, and fecal bile acids in dogs with gastrointestinal disease. PLoS One. (2019) 14:e0224454. doi: 10.1371/journal.pone.0224454 31671166 PMC6822739

[15] Sung CH Pilla R Marsilio S Chow B Zornow KA Slovak JE. Fecal concentrations of long-chain fatty acids, sterols, and unconjugated bile acids in cats with chronic enteropathy. Animals (Basel). (2023) 13:2753. doi: 10.3390/ani13172753, PMID: 37685017 PMC10486672

[16] Marsilio S Chow B Hill SL Ackermann MR Estep JS Sarawichitr B. Untargeted metabolomic analysis in cats with naturally occurring inflammatory bowel disease and alimentary small cell lymphoma. Sci Rep. (2021) 11:9198. doi: 10.1038/s41598-021-88707-5, PMID: 33911166 PMC8080598

[17] Marsilio S Ackermann MR Lidbury JA Suchodolski JS Steiner JM. Results of histopathology, immunohistochemistry, and molecular clonality testing of small intestinal biopsy specimens from clinically healthy client-owned cats. J Vet Intern Med. (2019) 33:551–8. doi: 10.1111/jvim.15455, PMID: 30820999 PMC6430868

[18] Watson H Mitra S Croden FC Taylor M Wood HM Perry SL. A randomised trial of the effect of omega-3 polyunsaturated fatty acid supplements on the human intestinal microbiota. Gut. (2018) 67:1974–83. doi: 10.1136/gutjnl-2017-314968, PMID: 28951525

[19] Jergens AE Crandell JM Evans R Ackermann M Miles KG Wang C. A clinical index for disease activity in cats with chronic enteropathy. J Vet Intern Med. (2010) 24:1027–33. doi: 10.1111/j.1939-1676.2010.0549.x, PMID: 20584141

[20] Kathrani A. Dietary and nutritional approaches to the Management of Chronic Enteropathy in dogs and cats. Vet Clin N Am Small Anim Pract. (2021) 51:123–36. doi: 10.1016/j.cvsm.2020.09.005, PMID: 33131914

[21] Keller SM Vernau W Moore PF. Clonality testing in veterinary medicine: a review with diagnostic guidelines. Vet Pathol. (2016) 53:711–25. doi: 10.1177/0300985815626576 26933096

[22] Tal M Verbrugghe A Gomez DE Chau C Weese JS. The effect of storage at ambient temperature on the feline fecal microbiota. BMC Vet Res. (2017) 13:256. doi: 10.1186/s12917-017-1188-z, PMID: 28821259 PMC5563020

[23] Marsilio S Pilla R Sarawichitr B Chow B Hill SL Ackermann MR. Characterization of the fecal microbiome in cats with inflammatory bowel disease or alimentary small cell lymphoma. Sci Rep. (2019) 9:19208. doi: 10.1038/s41598-019-55691-w, PMID: 31844119 PMC6914782

[24] Xia J Wishart DS. Web-based inference of biological patterns, functions and pathways from metabolomic data using MetaboAnalyst. Nat Protoc. (2011) 6:743–60. doi: 10.1038/nprot.2011.319, PMID: 21637195

[25] AlShawaqfeh MK Wajid B Minamoto Y Markel M Lidbury JA Steiner JM. A dysbiosis index to assess microbial changes in fecal samples of dogs with chronic inflammatory enteropathy. FEMS Microbiol Ecol. (2017) 93:136. doi: 10.1093/femsec/fix136, PMID: 29040443

[26] Vecchiato CG Pinna C Sung CH Borrelli de Andreis F Suchodolski JS Pilla R. Fecal microbiota, bile acids, sterols, and fatty acids in dogs with chronic enteropathy fed a home-cooked diet supplemented with coconut oil. Animals (Basel). (2023) 13:502. doi: 10.3390/ani13030502, PMID: 36766392 PMC9913398

[27] Masuoka H Shimada K Kiyosue-Yasuda T Kiyosue M Oishi Y Kimura S. Transition of the intestinal microbiota of cats with age. PLoS One. (2017) 12:e0181739. doi: 10.1371/journal.pone.0181739, PMID: 28813445 PMC5558916

[28] Blake AB Cigarroa A Klein HL Khattab MR Keating T van de Coevering P. Developmental stages in microbiota, bile acids, and clostridial species in healthy puppies. J Vet Intern Med. (2020) 34:2345–56. doi: 10.1111/jvim.15928, PMID: 33047396 PMC7694855

[29] Arnold C Pilla R Chaffin K Lidbury J Steiner J Suchodolski J. Alterations in the fecal microbiome and metabolome of horses with antimicrobial-associated diarrhea compared to antibiotic-treated and non-treated healthy case controls. Animals. (2021) 11:1807. doi: 10.3390/ani11061807, PMID: 34204371 PMC8235368

[30] Arnold CE Pilla R Chaffin MK Leatherwood JL Wickersham TA Callaway TR. The effects of signalment, diet, geographic location, season, and colitis associated with antimicrobial use or Salmonella infection on the fecal microbiome of horses. J Vet Intern Med. (2021) 35:2437–48. doi: 10.1111/jvim.16206, PMID: 34268795 PMC8478058

[31] Kathrani A Yen S Swann JR Hall EJ. The effect of a hydrolyzed protein diet on the fecal microbiota in cats with chronic enteropathy. Sci Rep. (2022) 12:2746. doi: 10.1038/s41598-022-06576-y, PMID: 35177696 PMC8854717

[32] Wang TY Liu M Portincasa P Wang DQH. New insights into the molecular mechanism of intestinal fatty acid absorption. Eur J Clin Investig. (2013) 43:1203–23. doi: 10.1111/eci.12161, PMID: 24102389 PMC3996833

[33] Lewis JD Chen EZ Baldassano RN Otley AR Griffiths AM Lee D. Inflammation, antibiotics, and diet as environmental stressors of the gut microbiome in pediatric Crohn’s disease. Cell Host Microbe. (2015) 18:489–500. doi: 10.1016/j.chom.2015.09.008, PMID: 26468751 PMC4633303

[34] Tang MS Poles J Leung JM Wolff MJ Davenport M. Inferred metagenomic comparison of mucosal and fecal microbiota from individuals undergoing routine screening colonoscopy reveals similar differences observed during active inflammation. Gut Microbes. (2015) 6:48–56. doi: 10.1080/19490976.2014.1000080, PMID: 25559083 PMC4615154

[35] Janeczko S Atwater D Bogel E Greiter-Wilke A Gerold A Baumgart M. The relationship of mucosal bacteria to duodenal histopathology, cytokine mRNA, and clinical disease activity in cats with inflammatory bowel disease. Vet Microbiol. (2008) 128:178–93. doi: 10.1016/j.vetmic.2007.10.014, PMID: 18054447

[36] Garraway K Johannes CM Bryan A Peauroi J Rossi G Zhang M. Relationship of the mucosal microbiota to gastrointestinal inflammation and small cell intestinal lymphoma in cats. J Vet Intern Med. (2018) 32:1692–702. doi: 10.1111/jvim.15291, PMID: 30084202 PMC6189339

[37] Sheehan D Moran C Shanahan F. The microbiota in inflammatory bowel disease. J Gastroenterol. (2015) 50:495–507. doi: 10.1007/s00535-015-1064-1 25808229

[38] Antharam VC McEwen DC Garrett TJ Dossey AT Li EC Kozlov AN. An integrated Metabolomic and microbiome analysis identified specific gut microbiota associated with fecal cholesterol and Coprostanol in *Clostridium difficile* infection. PLoS One. (2016) 11:e0148824. doi: 10.1371/journal.pone.0148824, PMID: 26871580 PMC4752508

[39] Filimoniuk A Daniluk U Samczuk P Wasilewska N Jakimiec P Kucharska M. Metabolomic profiling in children with inflammatory bowel disease. Adv Med Sci. (2020) 65:65–70. doi: 10.1016/j.advms.2019.12.009 31901795

[40] Jansson J Willing B Lucio M Fekete A Dicksved J Halfvarson J. Metabolomics reveals metabolic biomarkers of Crohn’s disease. PLoS One. (2009) 4:e6386. doi: 10.1371/journal.pone.0006386, PMID: 19636438 PMC2713417

[41] Guard BC Honneffer JB Jergens AE Jonika MM Toresson L Lawrence YA. Longitudinal assessment of microbial dysbiosis, fecal unconjugated bile acid concentrations, and disease activity in dogs with steroid-responsive chronic inflammatory enteropathy. J Vet Intern Med. (2019) 33:1295–305. doi: 10.1111/jvim.15493, PMID: 30957301 PMC6524081

[42] Wang S Martins R Sullivan MC Friedman ES Misic AM el-Fahmawi A. Diet-induced remission in chronic enteropathy is associated with altered microbial community structure and synthesis of secondary bile acids. Microbiome. (2019) 7:126. doi: 10.1186/s40168-019-0740-4, PMID: 31472697 PMC6717631

[43] Lai Y Xue J Liu CW Gao B Chi L Tu P. Serum metabolomics identifies altered bioenergetics, signaling cascades in parallel with Exposome markers in Crohn’s disease. Molecules. (2019) 24:449. doi: 10.3390/molecules24030449, PMID: 30691236 PMC6385106

[44] Crisi PE Luciani A di Tommaso M Prasinou P de Santis F Chatgilialoglu C. The fatty acid-based erythrocyte membrane Lipidome in dogs with chronic enteropathy. Animals (Basel). (2021) 11:2604. doi: 10.3390/ani11092604, PMID: 34573570 PMC8469057

[45] Scoville EA Allaman MM Adams DW Motley AK Peyton SC Ferguson SL. Serum polyunsaturated fatty acids correlate with serum cytokines and clinical disease activity in Crohn’s disease. Sci Rep. (2019) 9:2882. doi: 10.1038/s41598-019-39232-z, PMID: 30814550 PMC6393448

[46] Higueras C Rey AI Escudero R Díaz-Regañón D Rodríguez-Franco F García-Sancho M. Short-chain and Total fatty acid profile of Faeces or plasma as predictors of food-responsive enteropathy in dogs: a preliminary study. Animals (Basel). (2021) 12:89. doi: 10.3390/ani12010089 35011195 PMC8749849

[47] Guan S Jia B Chao K Zhu X Tang J Li M. UPLC-QTOF-MS-based plasma Lipidomic profiling reveals biomarkers for inflammatory bowel disease diagnosis. J Proteome Res. (2020) 19:600–9. doi: 10.1021/acs.jproteome.9b00440, PMID: 31821004

[48] Yuan SN Wang M Han JL Feng CY Wang M Wang M. Improved colonic inflammation by nervonic acid via inhibition of NF-κB signaling pathway of DSS-induced colitis mice. Phytomedicine. (2023) 112:154702. doi: 10.1016/j.phymed.2023.154702, PMID: 36764096

[49] Ramakers JD Mensink RP Schaart G Plat J. Arachidonic acid but not eicosapentaenoic acid (EPA) and oleic acid activates NF-kappaB and elevates ICAM-1 expression in Caco-2 cells. Lipids. (2007) 42:687–98. doi: 10.1007/s11745-007-3071-3, PMID: 17610002 PMC2039812

[50] Reinisch W Hung K Hassan-Zahraee M Cataldi F. Targeting endothelial ligands: ICAM-1/alicaforsen, MAdCAM-1. J Crohns Colitis. (2018) 12:S669–77. doi: 10.1093/ecco-jcc/jjy059, PMID: 29757363

[51] Castro-López J Ramis A Planellas M Teles M Pastor J. Cyclooxygenase-2 immunoexpression in intestinal epithelium and lamina propria of cats with inflammatory bowel disease and low grade alimentary lymphoma. BMC Vet Res. (2018) 14:158. doi: 10.1186/s12917-018-1486-0, PMID: 29764431 PMC5952374

[52] Galler AI Suchodolski JS Steiner JM Sung CH Hittmair KM Richter B. Microbial dysbiosis and fecal metabolomic perturbations in Yorkshire terriers with chronic enteropathy. Sci Rep. (2022) 12:12977. doi: 10.1038/s41598-022-17244-6, PMID: 35902689 PMC9334271

[53] Jia L Betters JL Yu L. Niemann-pick C1-like 1 (NPC1L1) protein in intestinal and hepatic cholesterol transport. Annu Rev Physiol. (2011) 73:239–59. doi: 10.1146/annurev-physiol-012110-142233, PMID: 20809793 PMC3965667

[54] Cuevas-Tena M Alegría A Lagarda MJ. Relationship between dietary sterols and gut microbiota: a review. Eur J Lipid Sci Technol. (2018) 120:1800054. doi: 10.1002/ejlt.201800054

[55] Cabral CE Klein MRST. Phytosterols in the treatment of hypercholesterolemia and prevention of cardiovascular diseases. Arq Bras Cardiol. (2017) 109:475–82. doi: 10.5935/abc.20170158 29267628 PMC5729784

[56] Zhang LS Davies SS. Microbial metabolism of dietary components to bioactive metabolites: opportunities for new therapeutic interventions. Genome Med. (2016) 8:46. doi: 10.1186/s13073-016-0296-x, PMID: 27102537 PMC4840492

[57] Reddy BS Wynder EL. Metabolic epidemiology of colon cancer: fecal bile acids and neutral 661 sterols in colon cancer patients and patients with adenomatous polyps. Cancer. (1977) 39:2533–9. doi: 10.1002/1097-0142(197706)39:6<2533::AID-CNCR2820390634>3.0.CO;2-X, PMID: 872053

[58] Gérard P Lepercq P Leclerc M Gavini F Raibaud P Juste C. Strain D8, the first cholesterol-reducing bacterium isolated from human feces. Appl Environ Microbiol. (2007) 73:5742–9. doi: 10.1128/AEM.02806-06, PMID: 17616613 PMC2074900

[59] Pilla R Gaschen FP Barr JW Olson E Honneffer J Guard BC. Effects of metronidazole on the fecal microbiome and metabolome in healthy dogs. J Vet Intern Med. (2020) 34:1853–66. doi: 10.1111/jvim.15871, PMID: 32856349 PMC7517498

[60] Duboc H Rajca S Rainteau D Benarous D Maubert MA Quervain E. Connecting dysbiosis, bile-acid dysmetabolism and gut inflammation in inflammatory bowel diseases. Gut. (2013) 62:531–9. doi: 10.1136/gutjnl-2012-302578, PMID: 22993202

[61] Li M Wang Q Li Y Cao S Zhang Y Wang Z. Apical sodium-dependent bile acid transporter, drug target for bile acid related diseases and delivery target for prodrugs: current and future challenges. Pharmacol Ther. (2020) 212:107539. doi: 10.1016/j.pharmthera.2020.107539, PMID: 32201314

[62] Veterinary Sciences *Free Full-Text Collaborative Metabolism: Gut Microbes Play a Key Role in Canine and Feline Bile Acid Metabolism*. (2024). Available at: https://www.mdpi.com/2306-7381/11/2/94. (Accessed February 26, 2024). 10.3390/vetsci11020094 PMC10892723 38393112

[63] Giaretta PR Rech RR Guard BC Blake AB Blick AK Steiner JM. Comparison of intestinal expression of the apical sodium-dependent bile acid transporter between dogs with and without chronic inflammatory enteropathy. J Vet Intern Med. (2018) 32:1918–26. doi: 10.1111/jvim.15332, PMID: 30315593 PMC6271328

[64] Jung D Fantin AC Scheurer U Fried M Kullak-Ublick GA. Human ileal bile acid transporter gene ASBT (SLC10A2) is transactivated by the glucocorticoid receptor. Gut. (2004) 53:78–84. doi: 10.1136/gut.53.1.78, PMID: 14684580 PMC1773940

[65] Ticho AL Malhotra P Dudeja PK Gill RK Alrefai WA. Intestinal absorption of bile acids in health and disease. Compr Physiol. (2019) 10:21–56. doi: 10.1002/cphy.c190007, PMID: 31853951 PMC7171925

[66] Liang Y Dong T Chen M He L Wang T Liu X. Systematic analysis of impact of sampling regions and storage methods on fecal gut microbiome and metabolome profiles. mSphere. (2020) 5:763. doi: 10.1128/msphere.00763-19, PMID: 31915218 PMC6952195

